# Phytochemicals from *Euclea natalensis* Modulate Th17 Differentiation, HIV Latency, and Comorbid Pathways: A Systems Pharmacology and Thermodynamic Profiling Approach

**DOI:** 10.3390/microorganisms13092150

**Published:** 2025-09-15

**Authors:** Ernest Oduro-Kwateng, Nader E. Abo-Dya, Mahmoud E. Soliman, Nompumelelo P. Mkhwanazi

**Affiliations:** 1 HIV Pathogenesis Programme, School of Laboratory Medicine and Medical Sciences, College of Health Science, University of KwaZulu-Natal, Durban 4041, South Africa; 223149553@stu.ukzn.ac.za; 2 Department of Pharmaceutical Chemistry, Faculty of Pharmacy, University of Tabuk, Tabuk 71491, Saudi Arabia; nabodya@ut.edu.sa; 3 Molecular Bio-Computation and Drug Design Research Group, School of Health Sciences, College of Health Science, University of KwaZulu-Natal, Durban 4041, South Africa

**Keywords:** *Euclea natalensis*, HIV/AIDS, network pharmacology, molecular modeling, Th17 differentiation, KEGG enrichment, MM/GBSA

## Abstract

HIV/AIDS remains a major global health challenge, with immune dysfunction, chronic inflammation, and comorbidities sustained by latent viral reservoirs that evade antiretroviral therapy. *Euclea natalensis*, a medicinal plant widely used in Southern African ethnomedicine, remains underexplored for its potential against HIV. An integrative systems pharmacology and molecular modeling framework was employed, including ADME profiling, target mapping, PPI network analysis, GO and KEGG pathway enrichment, BA-TAR-PATH analysis, molecular docking, MD simulations, and MM/GBSA calculations, to investigate the mechanistic roles of *E. natalensis* phytochemicals in HIV pathogenesis. Sixteen phytochemicals passed ADME screening and mapped to 313 intersecting host targets, yielding top ten hub genes with GO annotations in immune-metabolic, apoptotic, and nuclear signaling pathways. KEGG analysis revealed the enrichment of HIV-relevant pathways, including Th17 cell differentiation (hsa04659), PD-L1/PD-1 checkpoint (hsa05235), IL-17 signaling (hsa04657), HIF-1 signaling pathway (hsa04066), and PI3K-Akt (hsa04151). Lead phytochemicals, diospyrin and galpinone, strongly targeted key hub proteins (*NFκβ1*, *STAT3*, *MTOR*, *HSP90AA1*, and *HSP90AB1*), demonstrating favorable binding affinities, conformational stability, and binding free energetics compared to reference inhibitors. *E. natalensis* phytochemicals may modulate Th17 differentiation, HIV latency circuits, and comorbidity-linked signaling by targeting multiple host pathways, supporting their potential as multi-target therapeutic candidates for adjunct HIV/AIDS treatment and immunotherapy.

## 1. Introduction

Human immunodeficiency virus (HIV) remains a significant global health challenge, with an estimated 39.9 million people living with HIV (PLWH) infection as of 2023, with 630,000 mortalities [1]. Despite considerable progress in reducing morbidity and mortality through antiretroviral therapy (ART), a complete cure remains elusive in this patient population. Although ART suppresses viral replication and improves life expectancy, it does not completely eradicate the virus from latent reservoirs [2]. Furthermore, long-term ART use is associated with drug resistance, adverse metabolic effects, and immune dysfunction, which often predispose PLWH to opportunistic infections, complicating disease management and exacerbating healthcare burdens [3,4]. Natural products can serve as leads for the development of new antiviral agents or provide scaffolds for synthetic modifications, which can overcome the challenges of drug resistance and reduce cytotoxicity. When properly harnessed, their therapeutic potential can be significantly enhanced, leading to improved treatment outcomes for PLWH [5,6].

One of the critical unmet needs in HIV research is the development of novel therapeutic strategies that not only suppress viral replication but also restore immune function and eliminate latent reservoirs. Several therapeutic approaches are being explored, including latency-reversing agents (LRAs) (“shock-and-kill”), “block-and-lock” technique, broadly neutralizing antibodies, therapeutic vaccines, and immune checkpoint modulation [7,8]. The “shock-and-kill” strategy aims to eliminate latent HIV reservoirs by first reactivating the virus with LRAs and then targeting and killing the reactivated cells through the body’s immune system. The “block-and-lock” strategy aims to develop a functional cure by preventing HIV from reactivating, essentially locking the virus in a latent state. However, these approaches face obstacles, ranging from incomplete latency clearance to off-target effects and high costs [9,10]. In the landscape of HIV pathogenesis, accumulating evidence highlights the importance of targeting host immune responses, viral reservoirs, and dysregulated signaling pathways [11]. Consequently, there is growing interest in complementary or adjunctive interventions that are safe, effective, and accessible.

Natural products, particularly phytochemicals derived from medicinal plants, are gaining renewed interest in HIV cure research because of their structural diversity and multitarget activities. They offer a promising alternative or complementary strategy to existing therapies [5]. Among these, *Euclea natalensis* DC. (family Ebenaceae), traditionally used in African ethnomedicine to treat infections, inflammation, diabetes, and sexually transmitted diseases (STDs), has shown broad-spectrum biological activity via triterpenoids and naphthoquinones [12,13]. Phytochemical studies have identified key bioactive naphthoquinones in the root bark extracts, such as octahydroeuclein, 7-methyljuglone, and shinanolone, which exhibit competitive antifungal [14], antimycobacterial [15], and antibacterial properties [16]. However, the therapeutic application of *E. natalensis* in HIV treatment strategies remains largely unexplored. To the best of our knowledge, the only study on its anti-HIV activity reported the competitive activity of 7-methyljuglone against HIV-1 reverse transcriptase (RT) among other tested naphthoquinones, shinanolone, diospyrin, neodiospyrin, and isodiospyrin, in vitro [17]. Furthermore, studies have highlighted the potential of *E. natalensis* extracts to modulate key biological pathways in various diseases. For instance, methanol and dichloromethane extracts have been shown to ameliorate biochemical abnormalities in diabetic models by modulating the AMPK-GLUT4 pathway, suggesting a role in metabolic regulation [18]. Additionally, ethanolic extract exhibits immunomodulatory properties, enhancing T-helper 1 (Th1) cytokine production while suppressing T-helper 2 (Th2) responses, suggesting potential relevance in modulating immune hemostasis in microbial pathogenesis [19].

Network pharmacology, a modern systems biology framework, enables the mapping of complex interactions between phytochemicals and disease networks, moving beyond the traditional “one drug-one target” model. This approach is especially relevant in HIV cure research, where multifactorial host–pathogen interactions complicate therapeutic design. Although similar studies have applied network pharmacology to cancer [20], infectious diseases [21], and metabolic diseases [22], relatively few have leveraged this systems-level approach in HIV research [23]. In addition, integrative investigations focused on *E. natalensis* and HIV infection are currently lacking. This underscores the novelty and significance of our study in uncovering plant-based multi-target strategies for immune restoration and viral inhibition in the pathogenesis of HIV. Therefore, this study aimed to systematically investigate the therapeutic potential of *E. natalensis* in HIV pathogenesis by integrating systems pharmacology and molecular modeling approaches. Specifically, we combined absorption, distribution, metabolism, and excretion (ADME) profiling, host target mapping, protein–protein (PPI) analysis, gene ontology (GO) and Kyoto encyclopedia of genes and genomes (KEGG) enrichment, bioactive-target-pathway (BA-TAR-PATH) analysis, molecular docking, molecular dynamics (MD) simulations, and molecular mechanics/generalized Born surface area (MM/GBSA) binding free energy (BFE) calculations to identify and prioritize phytochemicals with favorable drug-like properties, uncover their putative host targets and HIV-relevant signaling pathways, and validate the stability and energetics of lead phytochemical–protein interactions.

Our enrichment analysis showed that hub genes targeted by *E. natalensis* phytochemicals are significantly associated with key immunoregulatory pathways, including Th17 cell differentiation, PD-1/PD-L1 signaling, and IL-17 signaling, suggesting the potential for the therapeutic modulation of disrupted immune homeostasis during HIV infection. Additionally, enriched annotations were observed in pathways related to HIV latency circuits and viral, oncogenic, and metabolic comorbidities, further highlighting the therapeutic relevance of these phytochemicals in addressing the multifaceted nature of HIV pathogenesis. The scope of our present study is limited to in silico analyses, providing mechanistic hypotheses for understanding the multi-target therapeutic potential of *E. natalensis*, paving the way for future experimental validation and drug development for HIV/AIDS management.

## 2. Materials and Methods

### 2.1. Collection of Bioactive Phytochemicals from Euclea natalensis

**Figure 1:**
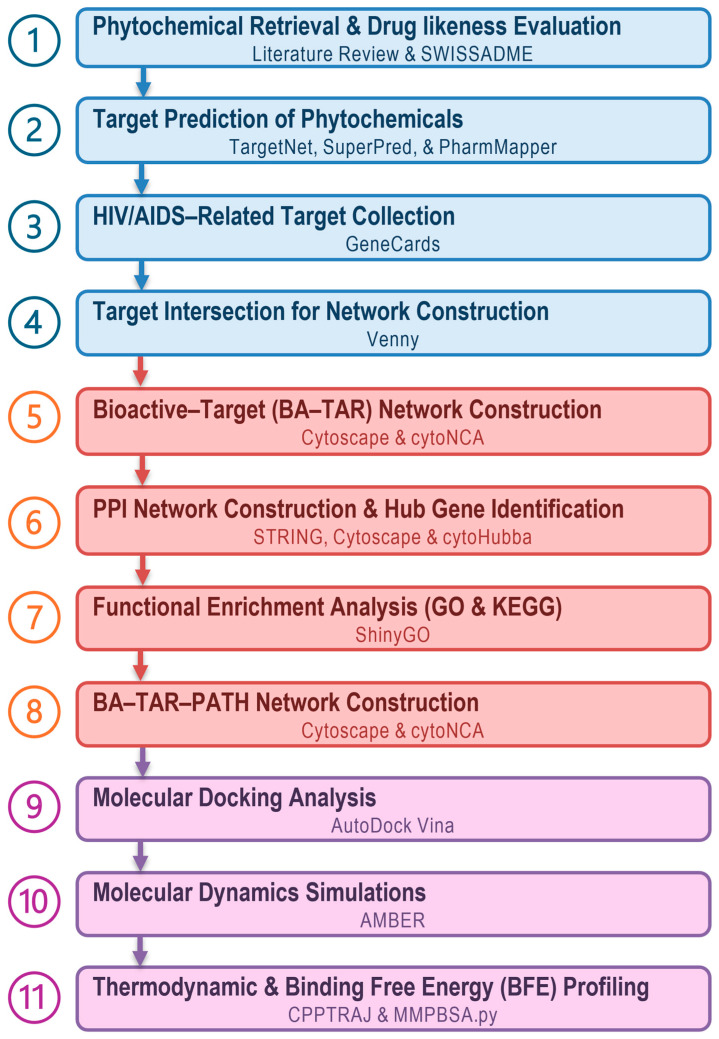
Schematic overview of the integrated computational workflow for investigating the anti-HIV/AIDS potential of *Euclea natalensis* phytochemicals.

The phytochemicals present in the root bark of *E. natalensis* were identified using a literature-based mining approach. A comprehensive search was conducted between 5 and 8 May 2025 using scientific databases including PubMed (https://pubmed.ncbi.nlm.nih.gov/) and Google Scholar (https://scholar.google.com/), focusing on publications that reported the isolation and characterization of phytochemicals from *E. natalensis* [14,15,16]. Priority was given to studies describing compounds with documented antiviral, immunomodulatory, and antimicrobial activities [12,13]. The chemical structures of the identified phytochemicals were retrieved and cross-referenced using the PubChem database (https://pubchem.ncbi.nlm.nih.gov/), accessed on 10 May 2025, from which the canonical SMILES notations and physicochemical properties were obtained [24]. For compounds lacking publicly available structures, two-dimensional (2D) chemical drawings were constructed using MarvinSketch v24.3.0, and the corresponding SMILES were generated [25]. The blueprint of the methodological workflow employed in this study is illustrated in Figure 1.

### 2.2. In Silico ADME Phytochemical Profiling

The pharmacokinetic profiles and drug-likeness of the phytochemicals were evaluated using SwissADME (http://www.swissadme.ch/), accessed on 11 May 2025. Lipinski’s Rule of Five (RO5) was applied to predict oral bioavailability (OB), permitting no more than one violation among the following criteria: molecular weight (MW) ≤ 500 Da, logP ≤ 5, hydrogen bond donors (HBD) ≤ 5, and hydrogen bond acceptors (HBA) ≤ 10. Additional parameters included topological polar surface area (TPSA) ≤ 140 Å^2^, gastrointestinal (GI) absorption rate, blood–brain barrier (BBB) permeability, bioavailability score (BS) ≥ 0.55, P-glycoprotein (P-gp) substrate status, and cytochrome P450 3A4 (CYP3A4) inhibition potential. Pan assay interference compounds (PAINS) alerts were recorded to avoid false-positive results in bioassays, and synthetic accessibility (SA) scores were used to estimate the practical feasibility of compound synthesis [26].

### 2.3. Prediction of Phytochemical Associated Human Targets

The canonical SMILES and SDF files of the identified compounds were queried across three databases: TargetNet (http://targetnet.scbdd.com/; accessed on 12 May 2025, probability cutoff ≥ 0.7) [27], SuperPred (https://prediction.charite.de/; accessed on 13 May 2025, probability threshold ≥ 70%) [28], and PharmMapper (http://www.lilab-ecust.cn/pharmmapper/; accessed on 13 May 2025, normalized fit score ≥ 0.7) [29]. Each compound was screened against the *Homo sapiens* proteome. The resulting target lists from each platform were compiled, and duplicate entries were removed. Protein targets were standardized and annotated using the UniProtKB database (https://www.uniprot.org/; accessed on 13 May 2025), ensuring consistent nomenclature by mapping to both the official protein and corresponding gene names [30].

### 2.4. Collection of HIV/AIDS-Associated Protein Targets

HIV/AIDS-related protein targets were identified by querying the comprehensive database of human genes, GeneCards (https://www.genecards.org/; accessed on 14 May 2025). Keywords included terms such as “*HIV*”, “*human immunodeficiency virus*”, “*HIV Infection*”, “*acquired immunodeficiency syndrome*”, “*AIDS*”, and “*HIV/AIDS*” to comprehensively capture disease-relevant genes. The resulting gene lists were combined, and duplicate entries were removed. Genes that lacked the corresponding UniProt identifiers were flagged for manual review and exclusion. The verified targets were cross-referenced and annotated using UniProtKB to ensure uniformity in protein/gene nomenclature.

### 2.5. Identification of HIV/AIDS-Related Host Targets

A comparative analysis was performed between the predicted targets of *Euclea natalensis* phytochemicals and curated HIV/AIDS host targets. The intersection of these datasets was determined using Venny 2.1.0 (https://bioinfogp.cnb.csic.es/tools/venny/), accessed on 15 May 2025. Overlapping targets were considered as the host targets potentially modulated by the phytochemicals for HIV/AIDS management.

### 2.6. Bioactive–Target (BA-TAR) Network Construction

A BA-TAR interaction network was constructed to visualize and explore the multi-component relationships between *Euclea natalensis* phytochemicals and the predicted overlapping HIV/AIDS-related host targets. The network was constructed using Cytoscape v3.10.3 (https://cytoscape.org; accessed on 15 May 2025). Each node in the network represents either a phytochemical or target protein, and the edges denote the predicted interactions between them. To evaluate the topological properties of the network, CytoNCA plug-in was employed to calculate degree, betweenness, and closeness centrality metrics. These measures respectively represent the number of direct connections of a node (degree), the extent to which a node lies on the shortest paths between other nodes (betweenness), and how close a node is to all other nodes in the network (closeness) [31]. Phytochemicals with high degree values were identified as key compounds, indicating potential multitarget activity and therapeutic relevance in modulating HIV/AIDS-associated host processes.

### 2.7. PPI Network Construction and Hub Gene Identification

To explore the potential interactions among HIV/AIDS-related host targets modulated by *Euclea natalensis* phytochemicals, a PPI network was constructed. The overlapping target genes were queried in the STRING database v12.0 (https://string-db.org/; accessed on 16 May 2025) with the organism restricted to *Homo sapiens* [32]. A medium confidence score threshold of 0.400 and a false discovery rate (FDR) stringency of 5% were applied to filter the interaction data. The resulting interaction network was imported into Cytoscape v3.10.3 for visualization and topological analysis. To identify key hub genes in the PPI network, we used the maximal clique centrality (MCC) algorithm implemented in the CytoHubba plugin in Cytoscape [33]. MCC quantifies node centrality based on the number and size of fully connected subgraphs (cliques), of which a node is a part. Therefore, protein targets with high MCC scores are considered central and potentially functionally significant in a PPI network. The top 10 hub genes with the highest MCC scores were selected as central targets, as they potentially play crucial roles in HIV/AIDS-related biological processes and serve as focal points for further functional and docking analyses.

### 2.8. GO and KEGG Pathway Enrichment Analysis

To elucidate the biological functions and signaling pathways associated with *Euclea natalensis* phytochemical targets in the context of HIV/AIDS, enrichment analysis was performed using ShinyGO v0.82 (https://bioinformatics.sdstate.edu/go/; accessed on 16 May 2025) [34]. The analysis was conducted using the top 10 hub genes identified from the PPI network based on MCC scoring. The input gene lists were analyzed under the species setting “*Homo sapiens*.” GO analysis was conducted to explore enriched biological processes (BPs), cellular components (CCs), and molecular functions (MFs). KEGG enrichment analysis was also performed to identify the most relevant signaling pathways that are potentially modulated by phytochemicals. Enriched terms were filtered based on FDR < 0.05, and the top 20 enriched GO terms and KEGG pathways were selected based on FDR-adjusted *p*-values and fold enrichment (FE) scores. The results were visualized as bubble charts displaying adjusted *p*-values and gene counts.

### 2.9. BAR-TAR-PATH Network Construction

To further illustrate the potential therapeutic mechanisms of *Euclea natalensis* phytochemicals in HIV-related host modulation, a BA-TAR-PATH network was constructed using Cytoscape v3.10.3 (https://cytoscape.org/; accessed on 17 May 2025). The network incorporated the bioactive compounds, their corresponding hub gene targets from the PPI analysis, and the top 20 enriched KEGG pathways. In the constructed network, each node represents a phytochemical, target gene, or KEGG pathway, and the edges represent the known or predicted interactions among them. The topological properties of the network were evaluated using the cytoNCA plugin, with the degree metric applied to quantify the number of direct connections associated with each target node [31]. In addition, an interactive network plot was generated to show the relationships between the top 20 enriched pathways. Two pathways are connected if they share 20% or more genes. Darker nodes represent more significantly enriched gene sets, larger nodes represent larger gene sets, and thicker edges represent more overlapping genes.

### 2.10. Molecular Docking Calculations

Molecular docking was performed to assess the binding affinity and interaction of selected *Euclea natalensis* phytochemicals with the HIV/AIDS-related human protein targets. To ensure biological relevance and network centrality, a refined subset of ligands and protein targets was selected by cross-referencing the top-ranked phytochemicals from the BA-TAR network topology (degree and betweenness centralities) with the hub genes identified from the PPI network analysis. These were further filtered based on their presence and high connectivity within the top 20 significantly enriched KEGG pathways, thereby prioritizing ligand-target pairs with functional prominence in both the BA-TAR and HIV/AIDS-related networks (BA-TAR-PATH). Three-dimensional (3D) crystal structures of the top-ranked hub targets were retrieved from the RCSB Protein Data Bank (PDB) (https://www.rcsb.org/; accessed on 18 May 2025). The selection criteria included X-ray crystallography-derived structures with a resolution ≤ 4.0 Å, presence of co-crystallized ligands, preference for human (*Homo sapiens*) proteins, and the highest-resolution structure when multiple entries existed for a given target.

The 3D structures of the selected *E. natalensis* phytochemicals were retrieved from the PubChem database or manually drawn and converted into 3D structures using MarvinSketch v24.3.0. Prior to docking, the compounds were energy-minimized using the general AMBER force field (GAFF) in Avogadro v1.2.0, and Gasteiger charges implemented in Chimera v1.19 were assigned. All the ligand files were saved in PDBQT format. Protein preparation was performed using Chimera v1.19, which involved the removal of water molecules and non-essential heteroatoms, separation of bound ligands, addition of polar hydrogens, and correction of missing side chains. The prepared target proteins were saved in PDB format, and the binding site coordinates were defined using AutoDock Tools v1.5.7, based on the position of the native co-crystallized ligands (Table S8). For protein structures lacking co-crystallized ligands, the most probable binding pockets were identified using CASTp 3.0 (http://sts.bioe.uic.edu/castp/; accessed on 19 May 2025) and DoGSiteScorer (https://proteins.plus/; accessed on 19 May 2025), and they were validated using the published structural literature to ensure accuracy. Furthermore, protonation states (pKa) of the prioritized phytochemicals were predicted using Epik 7 (Schrödinger Suite, 2023-4) in aqueous solvent at physiological pH (7.4 ± 0.5) [35] (Table S9).

Molecular docking simulations were performed using AutoDock Vina v1.1.2, incorporating a Genetic Algorithm (GA) for efficient conformational sampling and binding pose optimization. As a control step, the co-crystallized ligands were redocked to validate the docking protocol by evaluating the root mean square deviation (RMSD) between the experimental and predicted binding poses (Figure S1). To benchmark the docking performance of *E. natalensis* phytochemicals, molecular docking calculations included both co-crystallized ligands (where available) and known reference inhibitors: IMD 0354 (CID 5081913) for *NFκβ1* and 2-Methoxyestradiol (CID 66414) for *HIF1A*. This comparative strategy enabled the pharmacological evaluation of the binding affinity and interaction profiles of phytochemicals relative to those of the established modulators. The docking results were analyzed using BIOVIA Discovery Studio Visualizer 2024, with an emphasis on hydrogen bonding and hydrophobic contacts to identify ligand–target interactions of therapeutic relevance. To ensure that MD simulations focused on novel bioactive interactions and avoided redundancy, only the top-ranking phytochemical–protein complexes were advanced for the dynamic simulations.

### 2.11. MD Simulations

To evaluate the conformational stability and dynamic behavior of the top five high-scoring protein–ligand complexes, MD simulations were performed [36,37]. Simulations were performed using the AMBER18 suite (University of California, San Francisco, CA, USA), employing the PMEMD.CUDA module for GPU-accelerated calculations [38]. Ligands were parameterized using the ANTECHAMBER tool with RESP-calculated partial charges and GAFF. The pdb4amber utility was used to refine and prepare the protein structures for compatibility with AMBER topology. Each complex was neutralized with the appropriate counterions (Na^+^ or Cl^−^) and solvated in a TIP3P water box with a 12 Å buffer [39]. Energy minimization was performed in two stages: initial restrained minimization (500 kcal/mol Å^2^ restraint on solute atoms) for 2500 steps, followed by unrestrained minimization for 200 steps. The systems were then heated from 0 to 310 K over 50 ps in the NVT ensemble using a Langevin thermostat and 10 kcal/mol Å^2^ positional restraints. Equilibration was performed in the NPT ensemble, maintaining a pressure of 1 atm with a Berendsen barostat, and hydrogen bond constraints were applied via the SHAKE algorithm. Production MD simulations were conducted for 200 ns with a 2 fs time step at 310 K and 1 bar pressure under periodic boundary conditions. Trajectory analyses were performed using the CPPTRAJ module of AMBER, focusing on key structural parameters, including RMSD, root mean square fluctuation (RMSF), radius of gyration (RoG), and solvent-accessible surface area (SASA) [40]. Visualizations and structural inspections were performed using UCSF Chimera v1.19 and Discovery Studio Client 2024, and Origin 2018 was used for graphical analysis and statistical plotting.

### 2.12. BFE Computations

To quantitatively evaluate the binding affinities of *Euclea natalensis* phytochemicals with the selected HIV/AIDS-related host targets over time, BFE calculations were performed using the MM/GBSA method [41]. This approach integrates molecular mechanics energies with implicit solvation models to yield more accurate estimates of ligand–receptor binding energies compared to docking scores. Calculations were performed using the MMPBSA.py module from AMBER18, extracting 50,000 frames evenly spaced from the 200 ns MD trajectories of the top five protein–ligand complexes [42].

The MM/GBSA method computes the BFE (∆*G_bind_*) using the following equation:

(1) $${∆G}_{bind}=G_{complex}-G_{receptor}-G_{ligand}$$

This is expanded as

(2) $${∆G}_{bind}=E_{gas}+G_{sol}-T∆S$$

where

$E_{gas}=E_{int}+E_{vdw}+E_{ele}$; gas-phase energy composed of internal, van der Waals, and electrostatic interactions.

$G_{sol}=G_{GB}+G_{SA}$; solvation free energy, partitioned into polar (GB model) and nonpolar (SA model) components.

$G_{SA}=\gamma SASA$; nonpolar solvation, with γ = 0.0072 kcal/mol Å^2^ and SASA as the solvent-accessible surface area.

The entropic contribution, T∆S, was computed using normal mode analysis.

## 3. Results

### 3.1. In Silico ADME Evaluation of Phytochemical Ligands

ADME profiling of the screened phytochemicals revealed considerable diversity in their drug-likeness and pharmacokinetic properties (Table S1). Among the 17 compounds analyzed, most demonstrated favorable characteristics for OB and systemic exposure. Lipinski’s RO5 analysis showed that most compounds exhibited zero or only one violation, indicating good compliance with the established drug-like filters. Notably, compounds such as 5-hydroxy-4-methoxy-2-nathaldehyde, 7-methyljuglone, shinanolone, octahydroeuclein, and methylnaphthazarin showed full compliance with RO5, along with high GI absorption, good solubility (classified as “Soluble”), and a BS of 0.55, making them ideal candidates for further evaluation. In contrast, 20(29)-Lupene-3β-isoferulate was excluded from further analysis owing to significant pharmacokinetic limitations. It exhibited a high MW of 602.89 Da and logP of 6.23, and extremely poor water solubility (Log S = −10.94, classified as “Insoluble”). Additionally, it demonstrated low GI absorption and a poor BS of 0.17, along with a high SA of 6.75, collectively indicating limited druggability. Furthermore, several compounds, including diospyrin, euclanone, isodiospyrin, mamegakinone, natalenone, and neodiospyrin, exhibited moderate solubility, high GI absorption, and acceptable RO5 compliance despite being predicted as CYP3A4 inhibitors. Few compounds such as betulin, galpinone, lupeol, and β-sitosterol are poorly soluble with a low GI absorption rate. PAINS alerts were either absent or minimal across the compound set, further supporting the specificity of these phytochemicals for downstream biological evaluation. Moreover, most compounds showed SA scores below five, indicating a reasonable probability of synthetic feasibility. Table 1 shows the chemical properties of the 16 bioactive phytochemicals with optimal drug likeness.

### 3.2. Identification of Potential Targets of Euclea natalensis Phytochemicals in HIV Pathogenesis

**Figure 2:**
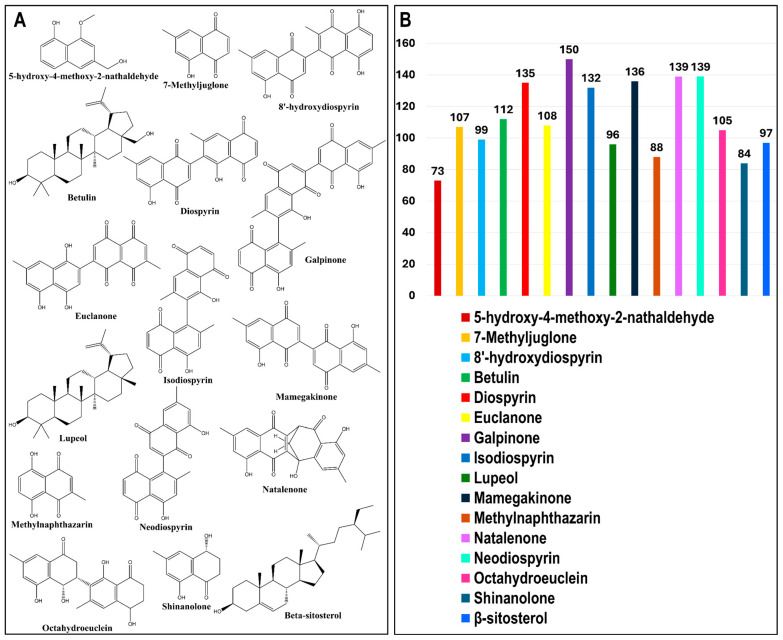
Chemical structures and target prediction profiles of the *Euclea natalensis* phytochemicals. (**A**) The 2D chemical structures of the 16 reported phytochemicals isolated from *E. natalensis* root bark. (**B**) Bar chart showing the number of predicted protein targets per compound identified using three target prediction platforms (TargetNet, SuperPred, and PharmMapper). Each color represents a distinct phytochemical.

**Figure 3:**
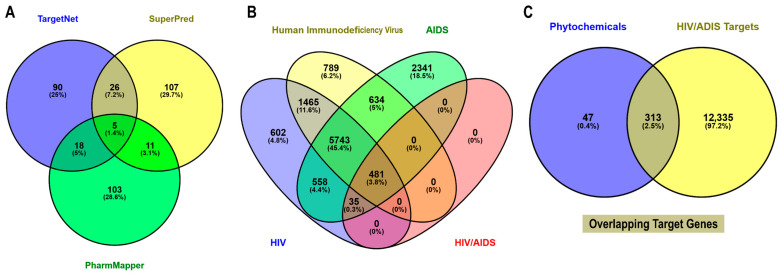
Target prediction and intersection of phytochemicals and HIV/AIDS-associated genes. (**A**) Venn diagram showing the overlap among the predicted protein targets of *Euclea natalensis* phytochemicals from TargetNet, SuperPred, and PharmMapper. (**B**) Overlapping HIV/AIDS-related target genes collected from GeneCards using four search terms: “*HIV*,” “*Human Immunodeficiency Virus*,” “*AIDS*,” and “*HIV/AIDS*.” (**C**) Common targets between predicted phytochemical targets and HIV/AIDS-related genes, resulting in 313 intersecting genes considered for further network analysis.

The phytochemicals of *E. natalensis* are clustered into three major scaffold types: naphthoquinone-based cores characterized by fused aromatic quinone systems, triterpenoid cores with pentacyclic lupane or oleanane skeletons, and steroidal cores represented by β-sitosterol, together with a few lower-molecular-weight compounds bearing simple phenolic or hydroxyaromatic frameworks (Figure 2A). Among the phytochemicals, galpinone (167), mamegakinone (164), neodiospyrin (158), isodiospyrin (156), and diospyrin (151) had the highest number of predicted targets, indicating their broad multi-target potential. In contrast, 5-hydroxy-4-methoxy-2-naphthaldehyde (96) and shinanolone (95) showed fewer predicted targets (Figure 2B). Target prediction for the 16 bioactive phytochemicals identified in *E. natalensis* was performed using TargetNet (139), SuperPred (149), and PharmMapper (137), yielding a total of 360 non-redundant human protein targets (Figure 3A). To identify HIV/AIDS-relevant targets, gene lists were retrieved from GeneCards using four keywords: “*HIV*” (8884), “*human immunodeficiency virus*” (9112), “*AIDS*” (9792), and “*HIV/AIDS*” (516), resulting in a merged and deduplicated total of 12,648 HIV/AIDS-associated human genes (Figure 3B).

A comparative intersection analysis using Venny 2.1 revealed 313 overlapping genes between the 360 phytochemical targets and the 12,648 HIV-related genes (Figure 3C). These intersecting genes were considered putative HIV/AIDS targets of *E. natalensis* phytochemicals and were selected for downstream network construction and enrichment analysis (Table S2).

### 3.3. BA-TAR Network Construction and Phytochemical Topological Analysis

**Figure 4:**
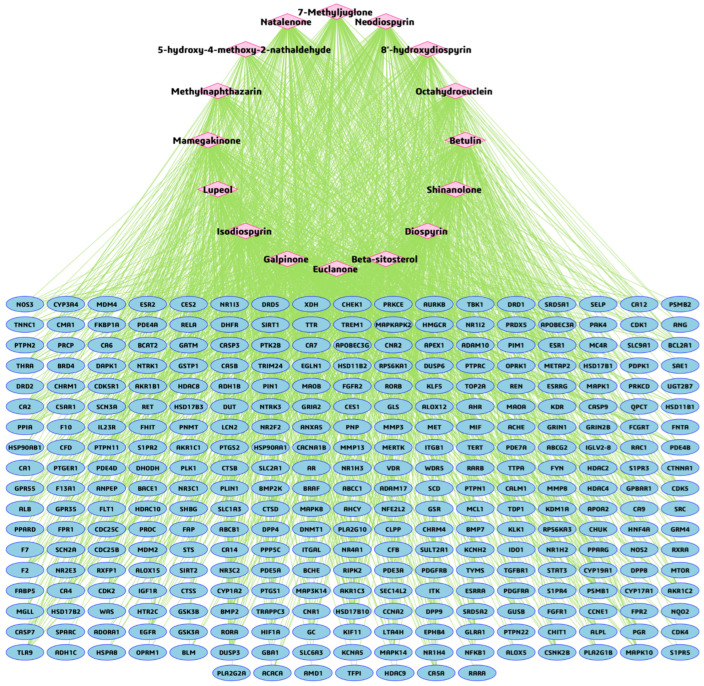
BAR-TAR interaction network of *Euclea natalensis* phytochemicals. The network representation illustrates the interactions (green edges) between 16 bioactive compounds from *E. natalensis* (pink nodes) and their predicted 313 human protein targets (blue nodes). The network emphasizes the polypharmacological profile of the phytochemicals, with several compounds interacting with multiple protein targets.

To investigate the multi-target interactions between *Euclea natalensis* phytochemicals and HIV/AIDS-related host proteins, a BA-TAR interaction network was constructed using Cytoscape v3.10.3. A total of 313 overlapping target genes and 16 phytochemicals were inputted to generate the network. The resulting network consisted of 329 nodes (representing phytochemicals and targets) and 1800 edges, indicating the predicted interactions (Figure 4). Network topological analysis revealed a characteristic path length of 1.000, a clustering coefficient of 0.000, and a network density of 0.017, indicating a sparsely connected but highly specific interaction structure. The network comprised a single connected component with no multi-edge node pairs or self-loops, suggesting well-defined compound–target relationships with minimal redundancy. To identify the core bioactive compounds, degree centrality (DC), betweenness centrality (BC), and closeness centrality (CC) metrics were computed using the cytoNCA plugin (Table 2). Among the 16 phytochemicals, galpinone exhibited the highest topological importance (DC = 150, BC = 12,264.043, CC = 0.490), followed by neodiospyrin (DC = 139), natalenone (DC = 136), mamegakinone (DC = 136), diospyrin (DC = 135), and isodiospyrin (DC = 132), each of which exhibited relatively high centrality metrics across BC and CC indices.

### 3.4. PPI Network and Hub Gene Analysis

**Figure 5:**
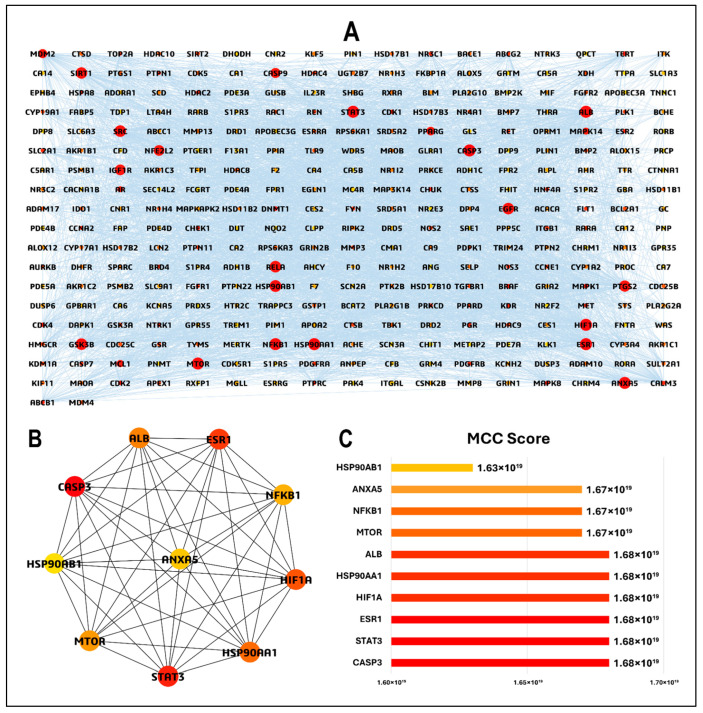
PPI network and hub gene analysis of overlapping HIV/AIDS-related targets. (**A**) PPI network constructed from the 313 overlapping targets of *Euclea natalensis* phytochemicals and HIV/AIDS-associated genes. Nodes represent protein targets, and the bigger red-highlighted nodes indicate the top-scoring hub genes. (**B**) Subnetwork of the top 10 hub genes ranked by MCC, visualized using CytoHubba. The node color (red to yellow) corresponds to the MCC score, with darker shades indicating higher centralities. (**C**) Bar plot of the top 10 hub genes sorted by MCC score. These genes are considered central regulators, suggesting their critical role in HIV/AIDS-related molecular pathogenesis.

To elucidate the molecular mechanisms underlying the therapeutic action of *Euclea natalensis* phytochemicals in HIV pathogenesis, a PPI network was constructed using the 313 overlapping targets between the predicted phytochemical targets and HIV/AIDS-associated genes. The network was generated using the STRING database (v12.0) under the “*Homo sapiens*” setting with a minimum required confidence score of 0.4. The resulting network comprised 308 nodes and 3649 edges, with an average node degree of 23.7, a local clustering coefficient of 0.494, and a highly significant PPI enrichment *p*-value < 1.0 × 10^−16^, indicating robust protein–protein associations beyond random expectation (Figure S2). The network was imported into Cytoscape v3.10.3 for enhanced visualization and topological analysis. The refined network retained all 308 nodes and 3649 edges, with a network density of 0.080, characteristic path length of 2.272, and network centralization of 0.400, suggesting a moderately connected and hierarchical topology. The network also displayed six connected components, with the largest comprising most nodes, highlighting the functional interconnectivity between the targets. To prioritize potential therapeutic targets, CytoHubba was used to calculate the MCC score for each node. Nodes were color-mapped from red (highest MCC) to yellow (lowest MCC), and the node size was scaled by the MCC score to visually identify the most influential hubs in the network (Figure 5A). The top 10 hub genes identified by MCC scoring were *CASP3*, *STAT3*, *ESR1*, *HIF1A*, *HSP90AA1*, *ALB*, *MTOR*, *NFκβ1*, *ANXA5*, and *HSP90AB1* (Figure 5B). The hub genes had MCC scores ranging from 1.63 × 10^19^ to 1.68 × 10^19^ (Figure 5C).

### 3.5. GO and KEGG Pathway Enrichment Analyses of Euclea natalensis-HIV/AIDS Hub Targets

#### 3.5.1. GO BP

**Figure 6:**
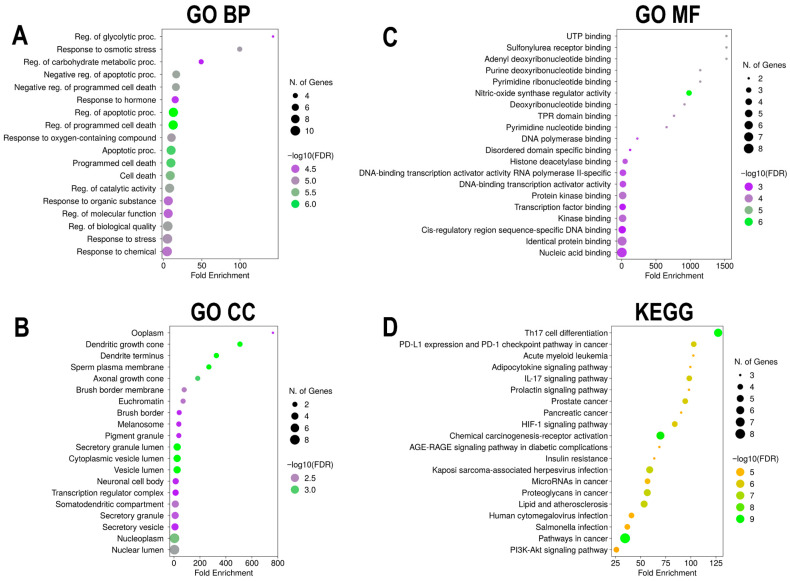
GO and KEGG pathway enrichment analysis of *Euclea natalensis* hub genes highlighting the top 20 enriched terms in bubble plots. (**A**) GO BP terms show enrichment in pathways, including regulation of glycolytic processes, stress response, and apoptotic regulation. (**B**) GO CC terms highlight localization in growth cones, plasma membranes, and secretory vesicles. (**C**) GO MF terms reveal enrichment in nucleotide binding, kinase binding, and transcription factor activity. (**D**) KEGG pathway analysis identified signaling pathways related to immunity, inflammation, infection, cancer, and metabolic disease, including Th17 differentiation, PD-1/PD-L1 checkpoint, IL-17 signaling, HIF-1 signaling, and PI3K-Akt signaling pathways. Dot sizes indicate the number of enriched genes per term, and the color scale represents statistical significance as −log_10_(FDR).

GO BP enrichment identified 1000 significant terms, with the top 20 primarily linked to cell death regulation, response to metabolic stress, and immune activation (Figure 6A). The top-ranked process was the regulation of glycolytic process (GO ID: GO:0006110), with the highest FE (143.01) and enrichment of hub genes *HIF1A*, *MTOR*, and *STAT3*. Other highly enriched terms included response to osmotic stress (GO:0006970), regulation of carbohydrate metabolism (GO:0006109), and response to hormone (GO:0009725). Multiple GO terms linked to apoptosis suppression and cell survival were also highly enriched, such as negative regulation of apoptotic process (GO:0043066), negative regulation of programmed cell death (GO:0043069), and regulation of cell death (GO:0010941), each involving seven, seven, and nine hub genes, respectively (Table S3).

#### 3.5.2. GO CC

Among the 109 enriched CC terms, the most significantly enriched were associated with protein-folding chaperone complexes, secretory pathways, and nuclear compartments (Figure 6B). Ooplasm (GO:1990917) had the highest FE (762.7), driven by *HSP90AB1*. Multiple terms related to vesicular and secretory structures, including secretory granule lumen (GO:0034774), cytoplasmic vesicle lumen (GO:0060205), and vesicle lumen (GO:0031983), were enriched (FE of 24.87, 24.67, and 24.54, respectively) and linked to *HSP90AA1*, *HSP90AB1*, *NFκβ1*, and *ALB*. Dendritic growth cone (GO:0044294) and axonal growth cone (GO:0044295) terms with FE scores of 508.47 and 183.05, respectively, were also enriched, involving both *HSP90* isoforms. Nuclear-associated components, such as euchromatin (GO:0000791) and transcription regulator complex (GO:0005667), were enriched with FE scores of 70.40 and 12.41, respectively, and involved *ESR1* and *HIF1A* (Table S4).

#### 3.5.3. GO MF

Of the 173 enriched MF terms, the top entries indicated involvement in nucleotide binding, transcriptional regulation, and kinase activity (Figure 6C). The top three enriched functions were UTP binding (GO:0002134), sulfonylurea receptor binding (GO:0017098), and adenyl deoxyribonucleotide binding (GO:0032558), each with an FE score of 1525.40. These terms were driven by the core chaperone proteins *HSP90AA1* and *HSP90AB1*. Several DNA- and transcription-related terms were also enriched, including DNA-binding transcription activator activity RNA polymerase II-specific (GO:0001228), and cis-regulatory region sequence-specific DNA binding (GO:0000987), with FE scores of 18.87 and 8.95, respectively. These functions involve transcriptional regulators such as *STAT3*, *HIF1A*, *NFκβ1*, and *ESR1*. Protein kinase binding (GO:0019901) and kinase binding (GO:0019900) were highly enriched, with FE scores of 15.48 and 13.88, respectively, involving *HSP90AA1*, *HSP90AB1*, *MTOR*, *STAT3*, and *ESR1* (Table S5).

#### 3.5.4. KEGG Pathway Enrichment Analysis

**Figure 7:**
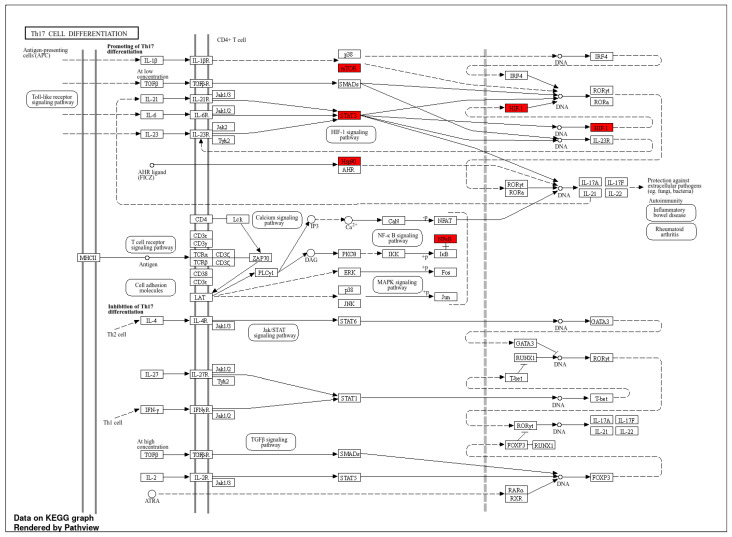
Integrated KEGG map of Th17 cell differentiation (hsa04659) highlighting hub genes targeted by *Euclea natalensis* phytochemicals. The pathway, rendered via Pathview, depicts the differentiation of naïve CD4^+^ T cells into Th17 cells, regulated by IL-6, IL-21, and IL-23 signaling via *STAT3*, and supported by *TGF-β*, *MTOR*, and *HIF-1*-dependent metabolic reprogramming. Hub genes modulated by *E. natalensis* (*MTOR*, *STAT3*, *HIF1A*, *NFκβ1*, *HSP90AA1*, and *HSP90AB1*) are highlighted in red. Solid arrows represent direct signaling events, while dashed arrows denote indirect or inferred interactions. Integrated modules including the Toll-like receptor (upper left), HIF-1 signaling (upper right), calcium signaling (center-left), and NFκ*β* and MAPK signaling (center-right) converge to regulate Th17 lineage specification. This map emphasizes the multilayered signaling complexity governing Th17 differentiation and the potential of *E. natalensis* phytochemicals to modulate this axis in the context of HIV immunopathogenesis.

A total of 123 KEGG pathways were significantly enriched. Many of the enriched pathways were immunological or oncogenic, reflecting host–pathogen interplay during HIV infection and its associated immune dysregulation (Figure 6D). The most enriched pathway was Th17 cell differentiation (KEGG ID: hsa04659; FE = 127.12), which involved six hub genes (*MTOR*, *HIF1A*, *HSP90AA1*, *HSP90AB1*, *NFκβ1*, and *STAT3*) (Figure 7). Other key immune pathways included PD-L1/PD-1 checkpoint pathway in cancer (hsa05235; FE = 102.84) and IL-17 signaling pathway (hsa04657; FE = 98.41). Oncogenic and immune-metabolic signaling pathways also featured prominently, such as HIF-1 signaling pathway (hsa04066), AGE-RAGE signaling in diabetic complications (hsa04933), and PI3K-Akt signaling pathway (hsa04151). These were enriched by combinations of *MTOR*, *STAT3*, *CASP3*, *HIF1A*, and *NFκβ1*. Viral infection-related pathways, such as Kaposi sarcoma-associated herpesvirus infection (hsa05167; FE = 58.97) and human cytomegalovirus infection (hsa05163; FE = 40.86), were enriched with *MTOR*, *NFκβ1*, *STAT3*, and *CASP3*. Notably, pathways in cancer (hsa05200; FE = 34.54) was the most gene-dense, involving eight hub genes, which is consistent with the hyperactivation of oncogenic signaling in chronic HIV infection and immune evasion (Table S6). Furthermore, the human immunodeficiency virus 1 (HIV-1) infection pathway (hsa05170) was significantly enriched with overlapping genes including *PAK4*, *CHEK1*, *CHUK*, *MAPK14*, *PTK2B*, *MTOR*, *TBK1*, *NFκβ1*, *MAPK1*, *MAPK8*, *MAPK10*, *RAC1*, *RELA*, *APOBEC3G*, *CALM1*, *CALM3*, *CASP3*, *CASP9*, *CDK1*, and *CDC25C* (Figure S3).

### 3.6. BA-TAR-PATH Network Construction

**Figure 8:**
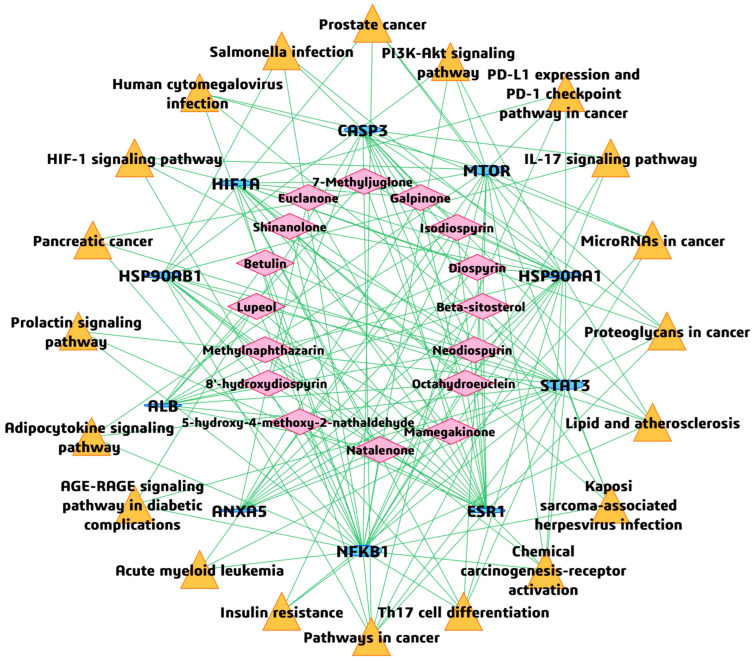
BA–TAR–PATH network of *Euclea natalensis* phytochemicals, hub proteins, and enriched KEGG pathways. The network illustrates interactions (green edges) among 16 phytochemicals (pink diamonds), 10 HIV-relevant hub genes (blue ellipses), and the top 20 enriched KEGG pathways (orange triangles). Dense interconnectivity highlights the multi-target effects and key regulatory roles of *NFκβ1*, *MTOR*, *HSP90AA1*, *HSP90AB1*, and *STAT3* in HIV-associated signaling.

To investigate the integrative relationships among key phytochemicals from *Euclea natalensis*, hub gene targets, and enriched biological pathways, a BA-TAR-PATH network was constructed using Cytoscape v3.10.3 (Figure 8). The network comprised 46 nodes (16 phytochemicals, 10 hub gene targets, and 20 KEGG pathways) and 165 edges, with a characteristic path length of 1.601 and a network diameter of 2, indicating strong interconnectedness and a short average distance between nodes. The average number of neighbors was 7.170, and the network density was 0.080, reflecting moderately high interaction complexity and redundancy within the therapeutic subnetwork. Topological analysis using the CytoNCA plugin revealed that several gene targets exhibited prominent centrality scores. Specifically, *NFκβ1* (DC = 27, BC = 492.572), *STAT3* (DC = 20, BC = 216.910), and *ESR1* (DC = 20, BC = 260.456) emerged as highly interconnected nodes, implicating their relevance in mediating the pharmacological effects of the phytochemicals. Other notable hub targets included *HSP90AA1* (DC = 19), *MTOR* (16), *CASP3* (16), *HIF1A* (15), and *HSP90AB1* (12), many of which are known regulators of immune responses and cell survival in HIV pathogenesis. With regards to the phytochemicals, natalenone and mamegakinone, both exhibited the highest DC (8), followed by octahydroeuclein (7), neodiospyrin (7), β-sitosterol (6), diospyrin (6), isodiospyrin (5), and galpinone (5), suggesting their broad-spectrum interaction across multiple hub genes and HIV/AIDS-relevant pathways. Importantly, these compounds and targets were also linked to the top KEGG pathways enriched in the study, such as Th17 cell differentiation (hsa04659), PD-L1 expression and PD-1 checkpoint pathway (hsa05235), IL-17 signaling pathway (hsa04657), HIF-1 signaling pathway (hsa04066), and PI3K-Akt signaling pathway (hsa04151). These pathways are implicated in immune regulation, T cell differentiation, apoptosis, and HIV-associated inflammation and immune evasion.

### 3.7. Inter-Pathway KEGG Network Connectivity

**Figure 9:**
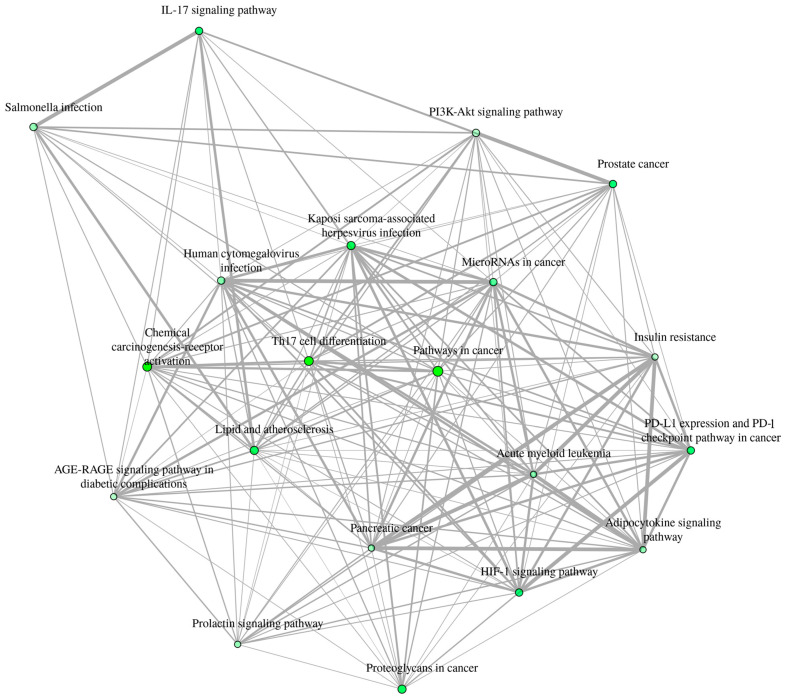
KEGG pathway–pathway enrichment network plot. Nodes represent the top 20 significantly enriched KEGG pathways. The node size corresponds to the size of each gene set, and the color (green) intensity reflects the statistical significance (FDR-adjusted *p*-values), with darker nodes indicating more significant enrichment. Edges represent ≥ 20% gene overlap between pathways; thicker edges indicate a higher gene overlap.

To explore the crosstalk between the enriched biological pathways, a KEGG pathway–pathway interaction network was generated based on the shared genes among the top 20 enriched KEGG terms (Figure 9). The network comprised 20 nodes and 165 edges. Each node represents a KEGG pathway, and an edge between any two nodes is defined by a ≥ 20% gene overlap threshold. Th17 cell differentiation (hsa04659) and pathways in cancer (hsa05200) were the most highly connected nodes, each showing multiple overlaps with other immune, viral, and oncogenic pathways, emphasizing the integrated nature of host immune signaling and virus-induced oncogenic transformation in HIV pathogenesis. Notably, the IL-17 signaling pathway (hsa04657), HIF-1 signaling pathway (hsa04066), and PD-L1 expression/PD-1 checkpoint pathway (hsa05235) were tightly linked, suggesting the concerted modulation of immune activation, inflammatory responses, and immune checkpoint signaling in the context of HIV infection. These associations further reinforce the relevance of the identified hub genes, such as *STAT3*, *MTOR*, *HIF1A*, and *NFκβ1*, as central mediators of immune dysregulation and HIV-related pathophysiology.

### 3.8. Molecular Docking Analysis

#### 3.8.1. Binding Affinity Calculations

To validate the interaction potential of prioritized ligand–target pairs, molecular docking simulations were performed between the top-ranking phytochemicals and HIV-associated hub proteins identified through the BA–TAR–PATH network and PPI analyses. Eight ligands with the highest DC (natalenone, mamegakinone, octahydroeuclein, neodiospyrin, β-sitosterol, diospyrin, isodiospyrin, and galpinone) were docked against eight functionally significant and topologically central protein targets: *NFκβ1*, *STAT3*, *ESR1*, *HSP90AA1*, *MTOR*, *CASP3*, *HIF1A*, and *HSP90AB1*. Binding affinities, expressed in kcal/mol, demonstrated robust interaction potential between the phytochemicals and their respective targets. To benchmark these results, co-crystallized ligands (where available), and reference inhibitors were included: IMD 0354 for *NFκβ1* (−6.5 kcal/mol) and 2-ME2 for *HIF1A* (−7.7 kcal/mol). Several phytochemical–protein complexes matched or exceeded these benchmarks, reinforcing their potential biological efficacy (Table 3). For *NFκβ1*, galpinone (−7.7 kcal/mol) and diospyrin (−7.6 kcal/mol) demonstrated stronger binding than IMD 0354. Similarly, for *HIF1A*, galpinone (−10.3 kcal/mol), natalenone (−8.8 kcal/mol), and diospyrin (−9.1 kcal/mol) all outperformed 2-ME2. Among all ligands, diospyrin showed the most potent binding to *HSP90AA1* (−11.6 kcal/mol) and *HSP90AB1* (−11.3 kcal/mol), surpassing the binding affinities of PU-11. Galpinone exhibited consistently strong binding across multiple key targets, including *STAT3* (−8.3 kcal/mol), *HSP90AA1* (−11.3 kcal/mol), *MTOR* (−10.3 kcal/mol), and *HSP90AB1* (−10.8 kcal/mol). Natalenone also demonstrated a significant affinity for *HSP90AA1* (−10.8 kcal/mol) and *HSP90AB1* (−10.5 kcal/mol). Meanwhile, isodiospyrin exhibited the strongest binding to *ESR1* (−9.5 kcal/mol), and mamegakinone showed notable interactions with both *MTOR* (−9.5 kcal/mol) and *CASP3* (−8.7 kcal/mol). Overall, the docking results corroborated the network-based prioritization, as the most strongly interacting ligands, diospyrin, galpinone, natalenone, isodiospyrin, and mamegakinone, were also among the most highly connected nodes within the BA–TAR–PATH network. These compounds demonstrated strong binding affinities to key HIV-associated proteins, such as *NFκβ1*, *MTOR*, *STAT3*, *HSP90AA1*, and *HSP90AAB1*, which are recurrent across 14, 13, 12, and 9 KEGG pathways, respectively.

#### 3.8.2. Protein–Ligand Interaction Analysis

**Figure 10:**
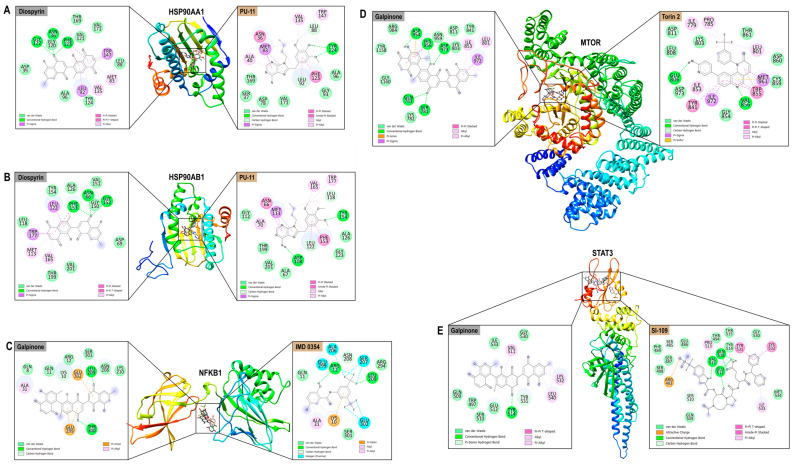
Comparative molecular docking analysis illustrating the binding interactions of the two top-ranked *Euclea natalensis* phytochemicals (diospyrin and galpinone, tan-colored) and their corresponding reference inhibitors (PU-11, IMD 0354, Torin 2, SI-109; slate gray) within the top-ranked hub HIV-associated host proteins. (**A**) *HSP90AA1*–Diospyrin vs. PU-11, (**B**) *HSP90AB1*–Diospyrin vs. PU-11, (**C**) *NFκβ1*–Galpinone vs. IMD 0354, (**D**) *MTOR*–Galpinone vs. Torin 2, and (**E**) *STAT3*–Galpinone vs. SI-109. The 2D interaction diagrams (left and right) show hydrogen bonds, hydrophobic contacts, and van der Waals interactions. Protein structures (center) are rainbow-colored by domain, with N-termini in blue and C-termini in red, illustrating the spatial orientation of ligand binding within the active site of each protein.

Detailed binding interaction profiling was conducted for the top five prioritized complexes: *HSP90AA1*–Diospyrin, *HSP90AB1*–Diospyrin, *MTOR*–Galpinone, *STAT3*–Galpinone, and *NFκβ1*–Galpinone. These were benchmarked against the respective reference inhibitors PU-11, Torin 2, SI-109, and IMD 0354. The phytochemicals formed extensive networks of stabilizing interactions within the binding sites of the HIV-relevant hub targets (Figure 10). *HSP90AA1*–Diospyrin complex formed strong conventional hydrogen bonds involving ASN36, GLY122, and PHE123. Notably, multiple π-interactions were observed with key residues, such as TRP147 and PHE123, along with van der Waals interactions stabilizing the ligand within the hydrophobic cleft. PU-11 formed diverse interaction patterns (Figure 10A). *HSP90AB1*–Diospyrin displayed a similar interaction profile with strong conventional hydrogen bonds (ASN66, GLY152, and PHE153), π–π stacking with TRP177 and PHE153, and a dense network of hydrophobic and van der Waals contacts. Similarly, PU-11 formed an extensive interaction footprint (Figure 10B). *NFκβ1*–Galpinone demonstrated hydrogen bonding with LEU209 and THR300 and π–anion interactions with GLU299 and GLU302. This binding mode closely paralleled that of the reference inhibitor IMD 0354, except for its peculiar fluorine halogen bonds with ALA206, SER207, GLU299, and GLU302 (Figure 10C). *MTOR*–Galpinone interaction involved five hydrogen bonds (GLN783, SER781, ASP954, HIS956, and ASP973), supported by π–π interactions with HIS956. Alkyl hydrophobic interactions involving LEU801 and ILE853 further anchored the compound to the active site. In contrast, Torin 2 engaged a broader spectrum of π-type hydrophobic interactions (Figure 10D). *STAT3*–Galpinone complex exhibited conventional hydrogen bonding with TYR514 and π-donor bonding with TYR531. The stabilizing interactions extended to hydrophobic contacts with LYS532 and LEU540, mirroring the interactions of the reference compound SI-109 (Figure 10E). Across all complexes, the interaction maps revealed that *E. natalensis* phytochemicals engaged similar binding residues and non-covalent interaction profiles compared to the benchmark inhibitors.

### 3.9. Thermodynamic Analyses of Top-Ranking Complexes

#### 3.9.1. Conformational Profiling of Hub Targets

**Figure 11:**
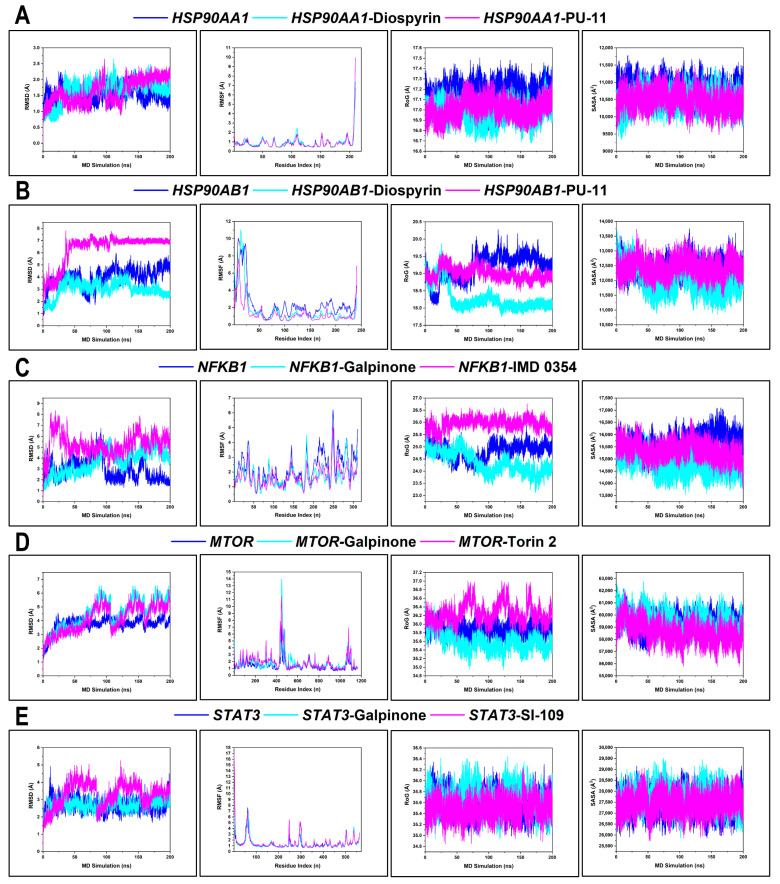
Superimposed line graphs illustrating comparative thermodynamic behaviors of (**A**) *HSP90AA1*, (**B**) *HSP90AB1*, (**C**) *NFκβ1*, (**D**) *MTOR*, and (**E**) *STAT3* in their apo forms (navy blue), bound to *Euclea natalensis* phytochemicals (diospyrin or galpinone, cyan), and reference inhibitors (magenta) across the 200 ns MD simulations. RMSD reflects overall structural stability, RMSF indicates per-residue flexibility, RoG denotes compactness of the protein fold, and SASA represents molecular exposure to solvent.

The conformational changes of the hub targets (*HSP90AA1*, *HSP90AB1*, *NFκB1*, *MTOR*, and *STAT3*) were assessed via RMSD, RMSF, RoG, and SASA (Table S10). The mean RMSD value of *HSP90AA1*, *HSP90AA1*–Diospyrin, and *HSP90AA1*–PU-11 was 1.53 Å, 1.67 Å, and 1.64 Å, respectively. Unbound *HSP90AA1* exhibited relatively stable RMSD fluctuations (~1.5 Å), with both ligated systems maintaining this stability throughout the simulation. However, PU-11 induced slightly higher RMSD excursions in the final 50 ns. The mean RMSF value of *HSP90AA1*, *HSP90AA1*–Diospyrin, and *HSP90AA1*–PU-11 was 0.89 Å, 0.92 Å, and 0.88 Å, respectively. RMSF plots confirmed minor per-residue fluctuations (< 2 Å), with the C-terminal region being more mobile. The average RoG value of *HSP90AA1*, *HSP90AA1*–Diospyrin, and *HSP90AA1*–PU-11 was 17.17 Å, 16.97 Å, and 17.01 Å, respectively. Thus, protein compactness remained tightly clustered (~17 Å) for all systems. Furthermore, the mean SASA value of *HSP90AA1*, *HSP90AA1*–Diospyrin, and *HSP90AA1*–PU-11 was 10,775.99 Å^2^, 10,467.41 Å^2^, and 10,467.00 Å^2^, respectively. The lower SASA in both ligand-bound systems indicates a more compact folding due to ligand-induced hydrophobic stabilization (Figure 11A).

The mean RMSD value of *HSP90AB1*, *HSP90AB1*–Diospyrin, and *HSP90AB1*–PU-11 was 3.99 Å, 3.02 Å, and 6.33 Å, respectively. The apo form fluctuated widely (3.0–6.5 Å), with PU-11 inducing fluctuations reaching over 7 Å, suggesting significant conformational drift. Diospyrin induced reduced backbone deviation. The mean RMSF value of *HSP90AB1*, *HSP90AB1*–Diospyrin, and *HSP90AB1*–PU-11 was 2.42 Å, 1.69 Å, and 1.31 Å, respectively. There were large spikes in flexible loop regions mostly for apo, while PU-11 suppressed these fluctuations than did diospyrin. The mean RoG value of *HSP90AB1*, *HSP90AB1*–Diospyrin, and *HSP90AB1*–PU-11 was 19.19 Å, 18.28 Å, and 19.00 Å, respectively. Protein fold was less compact for apo, reflecting the pronounced perturbations (>20 Å). The mean SASA value of *HSP90AB1*, *HSP90AB1*–Diospyrin, and *HSP90AB1*–PU-11 was 12,410.69 Å^2^, 12,021.46 Å^2^, and 12,442.97 Å^2^, respectively. SASA was markedly reduced by diospyrin compared to apo and PU-11, corroborating a diospyrin-induced structural tightening of *HSP90AB1* (Figure 11B).

The mean RMSD of *NFκβ1*, *NFκβ1*–Galpinone, and *NFκβ1*–IMD 0354 was 2.90 Å, 3.67 Å, and 5.25 Å, respectively. Unbound *NFκB1* exhibited the lowest RMSD profile compared to the ligand-bound systems. Notably, the IMD 0354 complex showed increased drift after ~150 ns. The mean RMSF of *NFκβ1*, *NFκβ1*–Galpinone, and *NFκβ1*–IMD 0354 was 2.29 Å, 1.72 Å, and 1.66 Å, respectively. RMSF values were highest in the apo system, reflecting ligand-induced per-residue stability. The mean RoG of *NFκβ1*, *NFκβ1*–Galpinone, and *NFκβ1*–IMD 0354 was 24.83 Å, 24.36 Å, and 25.95 Å, respectively. The mean SASA of *NFκβ1*, *NFκβ1*–Galpinone, and *NFκβ1*–IMD 0354 was 15,640.04 Å^2^, 14,776.18 Å^2^, and 15,350.44 Å^2^, respectively. RoG and SASA profiles further confirmed that galpinone induced structural compactness and decreased solvent exposure, reinforcing its stabilizing role (Figure 11C).

The mean RMSD of *MTOR*, *MTOR*–Galpinone, and *MTOR*–Torin 2 was 3.74 Å, 4.51 Å, and 4.16 Å, respectively. *MTOR* systems exhibited generally high RMSD values, consistent with its large, multi-domain architecture. Galpinone induced a higher fluctuation amplitude compared to Torin 2, particularly after 75 ns, suggesting ligand-induced destabilization. The mean RMSF of *MTOR*, *MTOR*–Galpinone, and *MTOR*–Torin 2 was 1.41 Å, 1.84 Å, 1.91 Å, respectively. The RMSF plot confirmed enhanced flexibility in the ligand-bound MTOR, particularly at residues in the 444–456 region. Despite these local fluctuations, the RoG values remained consistently tight across all systems, indicating that the global fold of *MTOR* was preserved. The mean RoG of *MTOR*, *MTOR*–Galpinone, and *MTOR*–Torin 2 was 35.88 Å, 35.59 Å, and 36.25 Å, respectively. SASA profiles were also similar, though the galpinone complex showed slightly elevated solvent exposure, supporting the inference of surface-exposed loop flexibility rather than large-scale unfolding. The mean SASA of *MTOR*, *MTOR*–Galpinone, and *MTOR*–Torin 2 was 59,263.54 Å^2^, 59,492.33 Å^2^, and 58,615.60 Å^2^, respectively (Figure 11D).

The mean RMSD of *STAT3*, *STAT3*–Galpinone, and *STAT3*–SI-109 was 2.70 Å, 2.69 Å, and 3.30 Å, respectively. Both the apo and *STAT3*–Galpinone systems stabilized rapidly and remained consistent throughout the 200 ns frame. In contrast, SI-109 could not maintain a stable RMSD convergence. The mean RMSF of *STAT3*, *STAT3*–Galpinone, and *STAT3*–SI-109 was 1.36 Å, 1.41 Å, and 1.56 Å, respectively. RMSF plots showed comparable residue-wise flexibility across all systems, with the highest fluctuations confined to the N-terminal end. Galpinone and SI-109 both slightly increased the flexibility of *STAT3*. RoG degrees remained tightly clustered with slight spikes observed for *STAT3*–Galpinone. The mean RoG of *STAT3*, *STAT3*–Galpinone, and *STAT3*–SI-109 was 35.58 Å, 35.68 Å, and 35.49 Å, respectively. Furthermore, the mean SASA of *STAT3*, *STAT3*–Galpinone, and *STAT3*–SI-109 was 27,414.40 Å^2^, 27,681.33 Å^2^, and 27,434.97 Å^2^, respectively. The SASA of the galpinone complex was marginally higher, potentially reflecting increased exposure of solvent-facing regions without compromising overall fold integrity (Figure 11E).

#### 3.9.2. BFE Profiling

BFE calculations of the top-ranked phytochemicals (diospyrin and galpinone) with hub targets (*HSP90AA1*, *HSP90AB1*, *NFκβ1*, *MTOR*, and *STAT3*) computed using MM/GBSA across 50,000 frames to complement thermodynamic stability analyses. The decomposition of van der Waals, electrostatic, solvation, and non-polar contributions revealed differential binding modes compared to reference inhibitors (Table 4). For *HSP90AA1*, diospyrin achieved a Δ*G_bind_* of −29.76 kcal/mol, whereas PU-11 bound more strongly at −38.91 kcal/mol. The interaction of diospyrin was primarily stabilized by van der Waals contributions (−42.38 kcal/mol) with moderate electrostatic terms (−10.48 kcal/mol), suggesting hydrophobic packing as the main driving force. A similar trend was observed for *HSP90AB1*, where diospyrin yielded a Δ*G_bind_* of −30.66 kcal/mol, weaker than PU-11 (−40.46 kcal/mol). Nevertheless, diospyrin reduced unfavorable polar solvation penalties compared to PU-11, reflecting its efficient hydrophobic accommodation within the *HSP90AB1* binding pocket. In the case of *NFκβ1*, galpinone displayed a Δ*G_bind_* of −26.85 kcal/mol, markedly stronger than IMD 0354 (−17.36 kcal/mol). The enhanced affinity stemmed from synergistic van der Waals (−47.89 kcal/mol) and electrostatic (−30.53 kcal/mol) interactions, accompanied by enhanced non-polar solvation energy (−5.41 kcal/mol). For *MTOR*, galpinone showed a Δ*G_bind_* of −28.88 kcal/mol, weaker than that of Torin 2 (−40.90 kcal/mol). While both ligands exhibited similar solvation free energy (~41 kcal/mol), Torin 2 gained substantially from its stronger electrostatic contribution (−34.48 kcal/mol) versus −24.96 kcal/mol for galpinone. Finally, for *STAT3*, galpinone exhibited a Δ*G_bind_* of −21.91 kcal/mol, while SI-109 bound significantly stronger at −70.01 kcal/mol. SI-109’s remarkable binding strength was largely driven by an unusually high electrostatic component (−306.50 kcal/mol) compensated by solvation penalties. In contrast, galpinone’s interaction was balanced between van der Waals (−32.43 kcal/mol) and moderate electrostatic contributions (−10.08 kcal/mol), indicating a more hydrophobic binding mode with less dependence on charged interactions.

## 4. Discussion

HIV pathogenesis is a complex and multifaceted process, involving the interplay between the virus and host immune responses. This study employed an integrative network pharmacology and molecular modeling pipeline to uncover the multi-target, pathway-level therapeutic relevance of *Euclea natalensis* phytochemicals in the landscape of HIV pathogenesis. To begin with, a set of 17 key phytochemicals was retrieved from the literature and subjected to ADME screening. The ADME findings confirmed that most of the phytochemicals exhibited favorable pharmacokinetic profiles, supporting their suitability for network pharmacology studies. 20(29)-Lupene-3β-isoferulate was excluded because of its multiple unfavorable properties, which compromises its drug-likeness. Although a few compounds presented potential metabolic liabilities, particularly CYP3A4 inhibition, their favorable absorption, acceptable bioavailability, and structural diversity warranted their inclusion in subsequent analyses [26]. To address any residual ADME limitations, advanced formulation strategies, such as liposomal encapsulation, nanoparticle delivery, nanohydrogels, or co-crystallization, may be considered to enhance bioavailability and therapeutic potential [6]. Subsequently, a set of 360 putative targets were identified for the 16 phytochemicals. These were intersected with 12,648 HIV/AIDS-related targets derived from GeneCards, yielding 313 overlapping genes with potential pharmacological relevance [43]. This intersection forms the therapeutic interface between *E. natalensis* constituents and HIV-associated host targets. BA-TAR network analysis revealed that phytochemicals such as galpinone, neodiospyrin, natalenone, mamegakinone, diospyrin, and isodiospyrin demonstrated high DC, indicating their broad and multi-target binding profiles. This multi-target behavior supports their potential for the systems-level modulation of HIV-related molecular networks. Furthermore, the integration of PPI network analysis with MCC-ranked hub genes highlighted key molecular regulators (*CASP3*, *STAT3*, *ESR1*, *HIF1A*, *HSP90AA1*, *ALB*, *MTOR*, *NFκβ1*, *ANXA5*, and *HSP90AB1*) that may underpin the multi-target therapeutic potential of *E. natalensis*. MCC outperforms other network analysis methods in identifying essential proteins by capturing their propensity to form dense clusters. Unlike methods that focus solely on local or global features, MCC integrates network density and community structure, making it more effective at detecting both highly connected nodes and low-degree, yet functionally important proteins within biological networks [33]. These targets are implicated in immune modulation, apoptosis, inflammation, viral replication, and cellular stress responses, which are mechanistically aligned with the immunopathogenesis and chronic persistence of HIV. Therefore, by modulating these interconnected pathways, *E. natalensis* phytochemicals may contribute to restoring immune homeostasis and disrupting viral propagation, offering a systems-level interventional strategy against HIV.

GO enrichment analysis provided insights into the possible mechanistic roles of *Euclea natalensis* phytochemicals in modulating HIV pathogenesis. The enriched BP suggest that these compounds may influence key immune-metabolic pathways, cellular stress responses, and apoptotic regulation—mechanisms commonly exploited by HIV to establish viral latency, evade immune surveillance, and drive chronic inflammation [44]. The CC annotations further reveal that many of the phytochemical-targeted proteins are localized in compartments integral to viral replication and immune modulation. These include the endoplasmic reticulum and cytoplasmic vesicles involved in protein folding and trafficking, secretory granules essential for cytokine export, and nuclear complexes that regulate transcription and chromatin remodeling, processes hijacked by HIV during infection and latency. Moreover, the enriched MF points to a broad spectrum of regulatory activity by *E. natalensis* constituents, including the modulation of transcription factor binding, signal transduction mediator activity, and PPIs, further supporting their systems-level relevance in HIV cure strategies [45].

Among the KEGG pathways enriched in this study, Th17 cell differentiation (hsa04659) emerged as the most significantly enriched and highly connected, intersecting with multiple hub proteins targeted by *Euclea natalensis* phytochemicals. This pathway plays a central role in maintaining immune equilibrium by regulating the differentiation of CD4^+^ T-cell subsets, particularly those involved in mucosal immunity. Th17 cells, predominantly located in gut-associated lymphoid tissue (GALT), secrete cytokines such as IL-17A, IL-17F, IL-21, and IL-22, which are essential for epithelial barrier integrity and antimicrobial defense [46,47]. In chronic HIV infection, Th17 cells are selectively depleted, contributing to microbial translocation, systemic inflammation, and immune dysfunction. Their loss is considered a hallmark of HIV-associated mucosal damage and disease progression [48,49]. Given the pivotal role of Th17 cells in mucosal immunity and their selective depletion during HIV infection, the modulation of this pathway could support mucosal restoration and systemic immune rebalancing. Hence, *E. natalensis* has the potential to restore Th17-mediated immune balance, suppress HIV-induced immune activation, and support mucosal immune recovery.

Our findings reveal that *Euclea natalensis* phytochemicals target several central regulators within the Th17 cell differentiation pathway (hsa04659), including *MTOR*, *STAT3*, *HIF1A*, *NFκβ1*, *HSP90AA1*, and *HSP90AB1*. *STAT3*, a master transcription factor, is indispensable for Th17 lineage commitment; it is activated downstream of IL-6, IL-21, and IL-23 signaling and induces RORγt, the lineage-defining transcription factor for Th17 cells [50]. *MTOR*, a metabolic checkpoint regulator, influences Th17 differentiation by promoting glycolysis and amino acid sensing required for CD4^+^ T-cell polarization [51]. *HIF1A*, stabilized under hypoxia, cooperates with *STAT3* and *MTOR* to favor Th17 differentiation while antagonizing regulatory T cell (Treg) development, which is a functional axis especially relevant in chronic HIV-driven inflammation and tissue hypoxia [51,52]. *NFκβ1*, a key component of the canonical *NFκβ* pathway, broadly modulates immune gene expression and inflammation and supports the expansion and cytokine production of Th17 cells [53]. *HSP90AA1* and *HSP90AB1*, molecular chaperones, are required for the stabilization and activation of client proteins such as *STAT3*, *HIF1A*, and *AKT*, which converge on the Th17 axis [54]. These chaperones not only support protein folding but also regulate inflammatory responses, and their inhibition has been shown to suppress Th17-mediated inflammation [55]. Previous studies have demonstrated that ethanolic shoot extracts of *Euclea natalensis* exhibit immunomodulatory activity in vitro, by enhancing Th1 cytokines (IL-2, IL-12, IFN-α) while downregulating Th2 cytokines such as IL-10 [19]. This Th1-skewing effect promotes cell-mediated immunity and reduces anti-inflammatory or immunosuppressive responses, creating a more favorable immune profile for pathogen clearance. In the in vivo models of *Mycobacterium tuberculosis* infection, this immunomodulatory action may have facilitated an effective pathogen-specific immune response, suggesting that *E. natalensis* may help restore immune balance in infectious disease contexts, including HIV. Although direct effects on Th17 cells are yet to be fully characterized, the enrichment of key upstream regulators, *STAT3*, *NFκβ1*, *HSP90*, and *MTOR*, by the phytochemicals suggests potential modulation of this lineage. Notably, phytomedicines such as *Vitis vinifera* (resveratrol) and *Curcuma longa* (curcumin) have been shown to inhibit *NFκβ* and *STAT3* signaling pathways. This inhibition leads to suppression of Th17 responses and contributes to the modulation of the Th17/Treg balance [56]. Furthermore, *Withania somnifera* (withaferin A) have shown similar Th17-modulatory activity through *HSP90* and *NFκβ* inhibition, offering precedent for such mechanisms in plant-based therapies for chronic immune dysregulation, including HIV/AIDS [57].

Furthermore, the enrichment of several KEGG pathway clusters underscores the multifaceted role of *Euclea natalensis* phytochemicals in HIV pathogenesis. Key oncogenic and immune-metabolic signaling pathways, including HIF-1 (hsa04066), AGE-RAGE in diabetic complications (hsa04933), and PI3K-Akt (hsa04151), highlight the complex interplay between hypoxia, oxidative stress, and chronic immune activation that HIV exploits to establish persistence and drive non-AIDS comorbidities such as metabolic syndrome and cancer [58]. These findings suggest that *E. natalensis* constituents may counteract HIV-induced cellular stress and immune exhaustion through metabolic reprogramming and redox modulation. Significantly enriched viral infection-related pathways, such as Kaposi sarcoma-associated herpesvirus (hsa05167) and human cytomegalovirus (hsa05163), reflect the heightened vulnerability of HIV-infected individuals to opportunistic infections [59]. The presence of *E. natalensis* targets within these pathways suggests broad-spectrum antiviral and immunomodulatory potential that may suppress HIV and co-pathogen replication while alleviating immune dysfunction. Crucially, Th17 cell differentiation (hsa04659) and pathways in cancer (hsa05200) emerged as the most connected nodes in the pathway-pathway network, reflecting central hubs of immune and oncogenic crosstalk in HIV/AIDS [60]. Moreover, the connectivity among IL-17 signaling (hsa04657), HIF-1 signaling (hsa04066), and PD-L1/PD-1 checkpoint (hsa05235) highlights a regulatory triad involved in inflammation, hypoxia adaptation, and immune evasion. Targeting this axis may disrupt the feedback loop sustaining HIV latency and immune exhaustion [52,61,62]. Overall, the pathway enrichment footprint supports a systems-level mechanism through which *E. natalensis* may restore immune homeostasis, suppress HIV pathogenesis, and reduce comorbidity burden. According to a study by Bati et al., 2024, treatment with *Euclea natalensis* leaf methanol and dichloromethane extracts significantly upregulated AMPK gene expression and enhanced GLUT4 protein expression in diabetic rat models [18]. This activation of the AMPK-GLUT4 axis promoted glucose uptake and metabolic homeostasis, supporting the antihyperglycemic effects observed. Notably, these metabolic regulatory effects align with the KEGG enrichment results from this study, namely the enrichment of the insulin resistance pathway (hsa04931) and PI3K-Akt signaling pathway (hsa04151). This further supports the notion that *E. natalensis* phytochemicals may possess therapeutic potential for addressing HIV-associated metabolic dysregulation. Moreover, *E. natalensis* phytochemicals were significantly enriched in the HIV-1 infection pathway (hsa05170), with overlapping genes converging on PI3K–Akt–MTOR signaling, NFκβ activation, MAPK cascades, calcium signaling, Toll-like receptor signaling, TNF signaling, and apoptotic pathways. These interconnected signaling modules are known to orchestrate key events in HIV replication dynamics, immune evasion, and latency maintenance. Therefore, by simultaneously engaging host immunoregulatory hubs and pro-viral effectors, the phytochemicals have the potential to exert multi-tiered control over HIV pathogenesis by circumventing chronic immune activation, modulating viral transcriptional reactivation, and restoring immune homeostasis.

Subsequently, a BA–TAR–PATH network was constructed, integrating PPI network and KEGG-based pathway enrichment. Topological analysis revealed that key gene targets such as *NFκβ1*, *STAT3*, *ESR1*, *HSP90AA1*, *MTOR*, *CASP3*, *HIF1A*, and *HSP90AB1* demonstrated high DCs and BCs, indicating their functional centrality and systemic influence in the HIV-related interactome. Moreover, these genes have been annotated in HIV latency dynamics [43]. In terms of phytochemical prioritization, natalenone, mamegakinone, octahydroeuclein, neodiospyrin, β-sitosterol, diospyrin, isodiospyrin, and galpinone exhibited the highest degree of connectivity across both network topology and target–pathway intersections. The engagement of these ligands across multiple targets and KEGG pathways reflects their multi-functional potential, an attribute desirable in combatting a multifactorial disease such as HIV/AIDS. Overall, docking results corroborated the network-based prioritization, as the most strongly interacting ligands, diospyrin, galpinone, natalenone, isodiospyrin, and mamegakinone, were also highly connected within the BA–TAR–PATH network. Their binding affinities to key HIV-associated proteins, *NFκβ1*, *MTOR*, *STAT3*, and *HSP90AA1*/*HSP90AB1*, were functionally prominent across 14, 13, 12, and 9 enriched KEGG pathways, respectively. This concordance reinforces their mechanistic relevance in modulating critical host pathways exploited during HIV pathogenesis, including immune activation, inflammation, and cellular stress responses.

Finally, the protein–ligand interaction profiles of diospyrin and galpinone reveal strong molecular interactions, including hydrogen bonding, hydrophobic interactions, and van der Waals contacts within functional regions of *HSP90* isoforms, *MTOR*, *STAT3*, and *NFκβ1*. The stability and diversity of interaction types observed in the complexes indicate that these ligands do not simply occupy the binding sites but may form energetically favorable and conformationally stable complexes. These findings were corroborated by reference inhibitor comparisons, where these ligands formed comparable interactions within functional domains, supporting their high-affinity binding behavior. MD simulations revealed that diospyrin and galpinone exert favorable target-specific thermodynamic effects. Diospyrin preserved the conformational stability of both *HSP90* isoforms, with notable reductions in RMSD, RoG, and SASA for *HSP90AB1* relative to PU-11, suggesting enhanced structural restraint and reduced solvent exposure. Similarly, galpinone stabilized *NFκB1*, improving folding compactness and solvent shielding slightly better than IMD 0354. While *MTOR* systems exhibited inherently high RMSD values owing to the protein’s domain-rich architecture, galpinone reduced residue-level fluctuations without perturbing global compactness, as reflected by favorable RMSF, RoG, and SASA values. In contrast, Torin 2 conferred slightly greater structural and hydrophobic stability. The *STAT3* dynamics further confirmed the compatibility of galpinone, with tight RMSD convergence and stable residue fluctuations maintained throughout the trajectory, in contrast to SI-109, which exhibited less stable binding. Furthermore, the BFE profiles underscore the ability of these phytochemicals to selectively modulate the hub targets. Diospyrin’s stabilization of *HSP90* isoforms through hydrophobic-driven binding may attenuate HSP90-mediated chaperoning of viral proteins and inflammatory kinases, thereby disrupting pathways that facilitate HIV replication and immune hyperactivation [63]. More notably, galpinone outperformed the reference inhibitor IMD 0354 at *NFκβ1*, a central transcriptional regulator of HIV-1 long terminal repeat (LTR) activity, suggesting potential to suppress viral transcriptional reactivation from latency [64]. Although *MTOR* and *STAT3* binding were weaker relative to Torin 2 and SI-109, respectively, the ability of galpinone to engage these nodes, which are key modulators of Th17 differentiation and immune exhaustion, points to a broader immunoregulatory effect, and this inhibitory effect suggests plausible latency stabilization [65]. Taken together, the thermodynamic favorability of diospyrin and galpinone supports their capacity to interfere with HIV latency circuits by simultaneously targeting molecular chaperones, transcriptional activators, and immune checkpoint regulators, highlighting their promise as natural multi-target agents in HIV cure strategies.

Experimental studies support the antiviral potential of related natural compounds. Notably, diospyrin, neodiospyrin, and isodiospyrin isolated from *Euclea natalensis* exhibited in vitro inhibitory activity against HIV-1 RT at concentrations of 25–50 µg/mL, with 7-methyljuglone showing higher activity at 6 µg/mL [17]. Similarly, betulinic acid derivatives, which are closely related triterpenoids also found in *E. natalensis*, have demonstrated efficacy as HIV-1 maturation inhibitors, with compounds such as bevirimat blocking Gag processing and reducing viral infectivity in vitro [66,67]. These findings provide experimental validation for the antiviral relevance of naphthoquinone and triterpenoid scaffolds, reinforcing the biological plausibility of our computational predictions and underscoring the potential of *E. natalensis* phytochemicals as multitarget agents against HIV pathogenesis. Naphthoquinones are a structurally diverse class of natural products with promising anti-HIV potential, acting at multiple stages of the viral life cycle. For instance, pyranonaphthoquinones and related analogs from *Ventilago harmandiana* inhibited HIV-1–induced syncytium formation with micromolar potency (EC_50_ = 9.9–47 µM), highlighting their ability to interfere with early events of viral spread [68]. Beyond entry, synthetic naphthoquinone Mannich bases have been identified as potent inhibitors of HIV-1 RT–associated RNase H, with lead compounds demonstrating low micromolar activity (IC_50_ = 2.8–3.1 µM) and favorable selectivity [69]. Likewise, trimeric naphthoquinone analogs of conocurvone strongly inhibited HIV-1 integrase (IN) (IC_50_ as low as 1.7–3 µM), underscoring their potential as scaffolds for IN-targeted therapy [70]. Together, these findings position naphthoquinones as multitarget antivirals with the unique advantage of disrupting HIV replication at distinct molecular checkpoints, offering a valuable framework for latency-modulating or combination therapeutic strategies against HIV. In addition to their direct antiviral effects, naphthoquinones modulate host signaling pathways that overlap with the hub targets identified in our study. Compounds such as plumbagin and shikonin suppress *NFκβ* activation by inhibiting Iκβ kinase (IKK) and preventing nuclear translocation of the *NFκβ* p50/p65 complex [71,72]. Plumbagin has also been shown to attenuate the PI3K/AKT/MTOR axis by reducing *MTOR* phosphorylation [73]and to inhibit *STAT3* TYR705 phosphorylation and transcriptional activity [74]. Moreover, *HSP90* isoforms (*HSP90AA1* and *HSP90AB1*), which act as molecular chaperones for *NFκβ*, *AKT*, and other HIV-supportive proteins, can be disrupted by inhibitors such as geldanamycin, leading to the degradation of client proteins and impairment of reactivation pathways [75]. Collectively, these findings underscore the inhibitory effects of naphthoquinones on multiple viral and host targets, including pathways that drive HIV persistence. This dual activity not only reinforces the experimental relevance of our in silico results but also highlights the potential of naphthoquinone scaffolds as latency-stabilizing agents in HIV cure research.

### Limitation of Study

Despite the strengths of this integrative systems pharmacology approach, our study is limited by its reliance on predicted target datasets and in silico simulations, which may not fully capture the complexity of host–pathogen interactions in HIV infection. The assumption of competitive inhibition for *E. natalensis* phytochemicals was based on docking and MD simulations, which were benchmarked against co-crystallized ligands and reference inhibitors with validated binding modes. However, while this provides a rationale for modeling their interactions at the canonical binding pockets of the key immune regulatory proteins involved in Th17 differentiation, computationally predicted binding affinities do not necessarily reflect functional inhibition. Therefore, the precise biological effects of these interactions, whether inhibitory, modulatory, or stabilizing, remain to be determined. Experimental validation through biochemical assays (e.g., enzyme inhibition, binding assays, and Western blot/ELISA) and HIV latency/reactivation models will be essential to confirm the proposed mechanisms. Overall, this study presents compelling in silico evidence for the multi-target pharmacological capacity of *E. natalensis* phytochemicals in modulating host proteins and pathways relevant to HIV progression. The convergence of high centrality scores, pathway enrichment, and binding energetics highlights a subset of compounds that merit experimental validation.

## 5. Conclusions

This study provides the first integrative systems pharmacology and molecular modeling evaluation of *Euclea natalensis* phytochemicals in HIV pathogenesis. The lead phytochemicals, diospyrin and galpinone, effectively modulated key hub targets, including *NFκβ1*, *STAT3*, *MTOR*, and *HSP90* isoforms, which are central to Th17 differentiation, HIV latency regulation, and chronic immune activation. Molecular docking, MD simulations, and MM/GBSA calculations confirmed strong binding affinities, high conformational stability, and favorable BFEs, which were comparable to those of the reference inhibitors. These results highlight the potential of *E. natalensis* phytochemicals as multi-target modulators of HIV-relevant signaling pathways and identify them as promising leads for adjunctive HIV therapy and immunomodulation.

Although these computational insights are encouraging, experimental validation is essential. Future studies should employ biochemical assays, real-time quantitative PCR (RT-qPCR), cytokine profiling of CD4^+^ T cells, and HIV latency reactivation or suppression models. Additionally, structure–activity relationship (SAR) optimization and formulation studies could enhance pharmacokinetics, supporting the translational development of these phytochemicals as therapeutic agents. Collectively, this study lays the foundation for the rational development of *E. natalensis* phytochemicals as multi-target interventions in HIV management.

## Figures and Tables

**Table 1:** Physicochemical properties of selected phytochemicals from *Euclea natalensis* root bark.

Phytochemical	Canonical SMILES	PubChem CID	MF	BS	RO5
5-hydroxy-4-methoxy-2-nathaldehyde	COC1=CC(CO)=CC2=C1C(O)=CC=C2	—	C_12_H_12_O_3_	0.55	0
7-Methyljuglone	CC1=CC2=C(C(=O)C=CC2=O)C(=C1)O	26905	C_11_H_8_O_3_	0.55	0
8′-hydroxydiospyrin	CC1=CC2=C(C(O)=C1)C(=O)C=C(C2=O)C1=C(C)C(=O)C2=C(C(O)=CC=C2O)C1=O	—	C_22_H_14_O_7_	0.55	0
Betulin	CC(=C)[C@@H]1CC[C@]2([C@H]1[C@H]3CC[C@@H]4[C@]5(CC[C@@H](C([C@@H]5CC[C@]4([C@@]3(CC2)C)C)(C)C)O)C)CO	72326	C_30_H_50_O_2_	0.55	1
Diospyrin	CC1=CC2=C(C(=C1)O)C(=O)C=C(C2=O)C3=C(C4=C(C=C3C)C(=O)C=CC4=O)O	308140	C_22_H_14_O_6_	0.55	0
Euclanone	CC1=CC2=C(C(=CC(=C2C(=C1)O)O)C3=CC(=O)C4=C(C3=O)C(=O)C(=CC4=O)C)O	633717	C_22_H_14_O_7_	0.55	0
Galpinone	CC1=CC2=C(C(=C1)O)C(=O)C(=CC2=O)C3=CC(=O)C4=C(C3=O)C(=C(C(=C4)C)C5=C6C(=O)C=CC(=O)C6=C(C=C5C)O)O	635975	C_33_H_20_O_9_	0.55	1
Isodiospyrin	CC1=CC2=C(C(=O)C=CC2=O)C(=C1C3=C4C(=O)C=CC(=O)C4=C(C=C3C)O)O	99298	C_22_H_14_O_6_	0.55	0
Lupeol	CC(=C)[C@@H]1CC[C@]2([C@H]1[C@H]3CC[C@@H]4[C@]5(CC[C@@H](C([C@@H]5CC[C@]4([C@@]3(CC2)C)C)(C)C)O)C)C	259846	C_30_H_50_O	0.55	1
Mamegakinone	CC1=CC2=C(C(=C1)O)C(=O)C(=CC2=O)C3=CC(=O)C4=C(C3=O)C(=CC(=C4)C)O	167673	C_22_H_14_O_6_	0.55	0
Methylnaphthazarin	CC1=CC(=O)C2=C(C=CC(=C2C1=O)O)O	271296	C_11_H_8_O_4_	0.55	0
Natalenone	[H]C1([H])C2C3=C(C(=O)C4=C(O)C=C(C)C=C4C3=O)C1(O)C1=C(C(O)=CC(C)=C1)C2=O	—	C_22_H_16_O_6_	0.55	0
Neodiospyrin	CC1=CC2=C(C(=C1)O)C(=O)C(=CC2=O)C3=C4C(=O)C=CC(=O)C4=C(C=C3C)O	16072922	C_22_H_14_O_6_	0.55	0
Octahydroeuclein	CC1=CC2=C([C@H]([C@H](CC2=O)C3=C(C4=C(C=C3C)C(CCC4=O)O)O)O)C(=C1)O	5273355	C_22_H_22_O_6_	0.55	0
Shinanolone	CC1=CC2=C(C(=O)CC[C@H]2O)C(=C1)O	5273357	C_11_H_12_O_3_	0.55	0
β-sitosterol	CC[C@H](CC[C@@H](C)[C@H]1CC[C@@H]2[C@@]1(CC[C@H]3[C@H]2CC=C4[C@@]3(CC[C@@H](C4)O)C)C)C(C)C	222284	C_29_H_50_O	0.55	1

PubChem Chemical identity (CID), molecular formula (MF), SMILES notation, bioactivity score (BS), and Lipinski’s Rule of Five (RO5) violations for selected phytochemicals isolated from the root bark of *E. natalensis* with reported bioactivity.

**Table 2:** Topological parameters of *Euclea natalensis* phytochemicals within the BA-TAR network ranked by DC.

	Phytochemical	DC	BC	CC
1	Galpinone	150	12,264.043	0.490
2	Neodiospyrin	139	7917.464	0.475
3	Natalenone	139	17,033.264	0.475
4	Mamegakinone	136	8877.140	0.471
5	Diospyrin	135	7291.925	0.469
6	Isodiospyrin	132	6207.231	0.465
7	Betulin	112	16,674.690	0.440
8	Euclanone	108	7403.342	0.436
9	7-Methyljuglone	107	4261.511	0.434
10	Octahydroeuclein	105	7981.093	0.432
11	8’-hydroxydiospyrin	99	6062.130	0.425
12	β-sitosterol	97	10,330.302	0.423
13	Lupeol	96	9744.878	0.422
14	Methylnaphthazarin	88	5105.912	0.414
15	Shinanolone	84	3258.515	0.409
16	5-hydroxy-4-methoxy-2-nathaldehyde	73	8500.559	0.399

**Table 3:** Docking scores (kcal/mol) of top-ranked phytochemicals with HIV/AIDS-associated hub proteins compared with co-crystallized ligands and reference inhibitors.

Ligand	Docking Score (kcal/mol)							
	*NFκβ1*	*STAT3*	*ESR1*	*HSP90AA1*	*MTOR*	*CASP3*	*HIF1A*	*HSP90AB1*
Natalenone	−7.4	−6.8	−5.7	−10.8	−8.0	−8.5	−8.8	−10.5
Mamegakinone	−7.2	−7.4	−6.0	−9.0	−9.5	−8.7	−8.7	−8.8
Octahydroeuclein	−6.6	−6.8	−5.9	−9.8	−8.7	−7.6	−7.9	−9.5
Neodiospyrin	−7.1	−7.3	−7.3	−10.8	−8.6	−8.5	−8.6	−10.4
β-sitosterol	−6.1	−5.9	−6.7	−9.7	−8.1	−7.5	−8.3	−9.5
Diospyrin	−7.6	−6.8	−5.4	−11.6	−9.4	−8.3	−9.1	−11.3
Isodiospyrin	−7.4	−6.9	−9.5	−10.5	−8.5	−8.0	−8.4	−9.9
Galpinone	−7.7	−8.3	−5.9	−11.3	−10.3	−9.2	−10.3	−10.8
Co-crystallized	-	SI-109	Estradiol	PU-11	Torin 2	MSI	-	PU-11
		−9.8	−10.6	−8.1	−11.6	−8.5		−8.0
Reference	IMD 0354	-	-	-	-	-	2-ME2	-
	−6.5						−7.7	

**Table 4:** BFE components (kcal/mol) of diospyrin and galpinone with hub targets (*HSP90AA1*, *HSP90AB1*, *NFκB1*, *MTOR*, and *STAT3*), calculated using the MM/GBSA method from 50,000 MD frames. Results are compared with reference ligands for each target, and the standard error of mean (SEM) is provided.

Protein–Ligand System	Energy Components (kcal/mol)						
	∆*E_vdW_*	∆*E_elec_*	∆*G_gas_*	∆*G_GB_*	∆*G_SA_*	∆*G_solv_*	∆*G_bind_*
*HSP90AA1*–Diospyrin	−42.38 ±0.10	−10.48 ±0.28	−52.85 ±0.27	28.12 ±0.21	−5.03 ±0.01	23.09 ±0.21	−29.76 ±0.11
*HSP90AA1*–PU-11	−46.52 ±0.09	−20.06 ±0.11	−66.59 ±0.13	33.58 ±0.09	−5.90 ±0.01	27.67 ±0.08	−38.91 ±0.09
*HSP90AB1*–Diospyrin	−44.52 ±0.07	−10.66 ±0.25	−55.17 ±0.25	29.89 ±0.20	−5.37 ±0.01	24.52 ±0.20	−30.66 ±0.09
*HSP90AB1*–PU-11	−45.99 ±0.09	−25.18 ±0.16	−71.17 ±0.17	36.63 ±0.12	−5.92 ±0.01	30.71 ±0.11	−40.46 ±0.10
*NFκβ1*–Galpinone	−47.89 ±0.14	−30.53 ±0.31	−78.41 ±0.39	56.97 ±0.32	−5.41 ±0.01	51.56 ±0.31	−26.85 ±0.13
*NFκβ1*–IMD 0354	−22.16 ±0.11	−42.15 ±0.34	−64.31 ±0.34	50.62 ±0.28	−3.67 ±0.01	46.95 ±0.27	−17.36 ±0.11
*MTOR*–Galpinone	−44.77 ±0.13	−24.96 ±0.20	−69.73 ±0.24	45.39 ±0.20	−4.54 ±0.01	40.85 ±0.21	−28.88 ±0.14
*MTOR*–Torin 2	−47.36 ±0.11	−34.48 ±0.18	−81.84 ±0.21	45.52 ±0.16	−4.58 ±0.01	40.94 ±0.17	−40.90 ±0.11
*STAT3*–Galpinone	−32.43 ±0.15	−10.08 ±0.13	−42.51 ±0.20	24.63 ±0.12	−4.03 ±0.02	20.60 ±0.11	−21.91 ±0.13
*STAT3*–SI-109	−46.35 ±0.18	−306.50 ±1.2	−352.85 ±1.2	289.84 ±0.99	−7.00 ±0.01	282.84 ±0.99	−70.01 ±0.33

Electrostatic energy (∆*E_elec_*), van der Waals energy (∆*E_vdW_*), gas-phase energy (∆*G_gas_*), polar solvation energy (∆*G_GB_*), non-polar solvation energy (∆*G_SA_*), total solvation free energy of polar and non-polar components (∆*G_solv_*), and total free binding energy (∆*G_bind_*).

## Data Availability

The original contributions presented in this study are included in the article/Supplementary Materials. Further inquiries can be directed to the corresponding authors.

## Supplementary Materials

The following supporting information can be downloaded at: https://www.mdpi.com/article/10.3390/microorganisms13092150/s1, Figure S1: Validation of molecular docking protocol via redocking of co-crystallized ligands using AutoDock Vina. Figure S2: PPI network of overlapping targets between *Euclea natalensis* phytochemicals and HIV/AIDS-associated genes. Figure S3: Integrated KEGG map of Human immunodeficiency virus type 1 (HIV-1) infection (hsa05170) highlighting overlapping genes targeted by *Euclea natalensis* phytochemicals. Table S1: Predicted ADME and drug-likeness properties of phytochemicals from *Euclea natalensis* root bark based on SwissADME analysis. Table S2: UniProt identifiers and gene/protein annotations for the 313 overlapping targets between *Euclea natalensis* phytochemicals and HIV/AIDS-associated genes. Table S3: The top 20 enriched GO biological processes (BPs) associated with the hub genes targeted by *Euclea natalensis* phytochemicals. Table S4: The top 20 enriched GO cellular components (CCs) associated with the hub genes targeted by *Euclea natalensis* phytochemicals. Table S5: The top 20 enriched GO molecular functions (MFs) associated with the hub genes targeted by *Euclea natalensis* phytochemicals. Table S6: The top 20 enriched KEGG pathways associated with hub genes targeted by *Euclea natalensis* phytochemicals. Table S7: Topological parameters of key nodes, including hub genes and *Euclea natalensis* phytochemicals, in the BA-TAR-PATH network construction, ranked by degree centrality. Table S8: Protein targets, PDB IDs, resolutions, and docking coordinates of the hub genes in the BA-TAR-PATH network analysis selected for molecular docking. Table S9: Predicted ionization states and pKa values for the 8 key *E. natalensis* phytochemicals at physiological pH (7.4 ± 0.5), calculated using Epik (Schrödinger Suite 2023-2). For each ionizable atom, the predicted acidic or basic pKa, penalty (kcal/mol), and most probable net charge are reported. Table S10: Thermodynamic descriptors of protein–ligand complexes based on 200 ns MD simulations.

## Abbreviations

ADME	Absorption, distribution, metabolism, and excretion
AIDS	Acquired immunodeficiency syndrome
ART	Antiretroviral therapy
BA-TAR	Bioactive-target
BA-TAR-PATH	Bioactive-target-pathway
BFE	Binding free energy
BP	Biological process
CC	Cellular component
GO	Gene ontology
GALT	Gut-associated lymphoid tissue
HIF-1	Hypoxia-inducible factor 1
HIV	Human immunodeficiency virus
KEGG	Kyoto encyclopedia of genes and genomes
IL	Interleukin
MCC	Maximal clique centrality
MD	Molecular dynamics
MM/GBSA	Molecular mechanics/generalized Born surface area
MF	Molecular function
PPI	Protein–protein interaction
PD-1	Programmed cell death protein 1
PD-L1	Programmed death ligand 1
RMSD	Root mean square deviation
RMSF	Root mean square fluctuation
RoG	Radius of gyration
SASA	Solvent accessible surface area
Th17	T helper 17 (CD4+ T-cell subtype)

## References

[1] Chen Y., Li A.D., Yang Y., Lu J., Xu Y., Ji X., Wu L., Han L., Zhu B., Xu M. (2025). Global, Regional and National Burden of HIV/AIDS among Individuals Aged 15–79 from 1990 to 2021. AIDS Res. Ther..

[2] Pasternak A.O., Berkhout B. (2023). HIV Persistence: Silence or Resistance?. Curr. Opin. Virol..

[3] Vaillant A.A.J., Naik R. (2023). HIV-1–Associated Opportunistic Infections.

[4] Chawla A., Wang C., Patton C., Murray M., Punekar Y., de Ruiter A., Steinhart C. (2018). A Review of Long-Term Toxicity of Antiretroviral Treatment Regimens and Implications for an Aging Population. Infect. Dis. Ther..

[5] Mandal A., Biswas D., Hazra B. (2020). Natural Products from Plants with Prospective Anti-HIV Activity and Relevant Mechanisms of Action. Stud. Nat. Prod. Chem..

[6] Wang J., Guo N., Hou W. (2023). Antiviral Drug Carriers for Human Immunodeficiency Virus. Nano Trends.

[7] Pellaers E., Denis A., Debyser Z. (2024). New Latency-Promoting Agents for a Block-and-Lock Functional Cure Strategy. Curr. Opin. HIV AIDS.

[8] Kim Y., Anderson J.L., Lewin S.R. (2018). Getting the “Kill” into “Shock and Kill”: Strategies to Eliminate Latent HIV. Cell Host Microbe.

[9] Maina E.K., Adan A.A., Mureithi H., Muriuki J., Lwembe R.M. (2020). A Review of Current Strategies Towards the Elimination of Latent HIV-1 and Subsequent HIV-1 Cure. Curr. HIV Res..

[10] Sadowski I., Hashemi F.B. (2019). Strategies to Eradicate HIV from Infected Patients: Elimination of Latent Provirus Reservoirs. Cell. Mol. Life Sci..

[11] Ward A.R., Mota T.M., Jones R.B. (2021). Immunological Approaches to HIV Cure. Semin. Immunol..

[12] Maroyi A. (2017). Review of Ethnomedicinal Uses, Phytochemistry and Pharmacological Properties of *E. natalensis* A.DC. Molecules.

[13] Taye A.D., Bizuneh G.K., Kasahun A.E. (2023). Ethnobotanical Uses, Phytochemistry and Biological Activity of the Genus Euclea: A Review. Front. Pharmacol..

[14] Lall N., Weiganand O., Hussein A.A., Meyer J.J.M. (2006). Antifungal Activity of Naphthoquinones and Triterpenes Isolated from the Root Bark of *E. natalensis*. S. Afr. J. Bot..

[15] van der Kooy F., Meyer J.J.M., Lall N. (2006). Antimycobacterial Activity and Possible Mode of Action of Newly Isolated Neodiospyrin and Other Naphthoquinones from *E. natalensis*. S. Afr. J. Bot..

[16] Weigenand O., Hussein A.A., Lall N., Meyer J.J.M. (2004). Antibacterial Activity of Naphthoquinones and Triterpenoids from *E. natalensis* Root Bark. J. Nat. Prod..

[17] Tshikalange T.E., Lall N., Meyer J.J.M., Mahapatra A. (2007). In Vitro HIV-1 Reverse Transcriptase Inhibitory Activity of Naphthoquinones and Derivatives from *E. natalensis*. S. Afr. J. Bot..

[18] Bati K., Baeti P.B., Gaobotse G., Kwape T.E. (2024). Leaf Extracts of *E. natalensis* A.D.C Ameliorate Biochemical Abnormalities in High-Fat-Low Streptozotocin-Induced Diabetic Rats through Modulation of the AMPK-GLUT4 Pathway. Egypt. J. Basic Appl. Sci..

[19] Lall N., Kumar V., Meyer D., Gasa N., Hamilton C., Matsabisa M., Oosthuizen C. (2016). In Vitro and In Vivo Antimycobacterial, Hepatoprotective and Immunomodulatory Activity of *E. natalensis* and Its Mode of Action. J. Ethnopharmacol..

[20] Jain N.K., Chandrasekaran B., Khazaleh N., Jain H.K., Lal M., Joshi G., Jha V. (2025). Computational Network Pharmacology, Molecular Docking, and Molecular Dynamics to Decipher Natural Compounds of *Alchornea laxiflora* for Liver Cancer Chemotherapy. Pharmaceuticals.

[21] Gois Leandro C., Senesi P., Singh Grewal A., Yan Z., Wei C. (2019). Integrated Bioinformatics and in Silico Approaches Reveal the Biological Targets and Molecular Mechanisms of 1,25-Dihydroxyvitamin D against COVID-19 and Diabetes Mellitus. Front. Nutr..

[22] Akoonjee A., Lukman H.Y., Ajao A.A., Uthman T.O., Sabiu S. (2024). A Network Pharmacology- and Molecular Dynamics Simulation-Based Bioprospection of *Khaya grandifoliola* C. DC. for diabetes care. J. Biomol. Struct. Dyn..

[23] Shao C., Wang H., Sang F., Xu L. (2022). Study on the Mechanism of Improving HIV/AIDS Immune Function with Jian Aikang Concentrated Pill Based on Network Pharmacology Combined with Experimental Validation. Drug Des. Dev. Ther..

[24] Kim S., Thiessen P.A., Bolton E.E., Chen J., Fu G., Gindulyte A., Han L., He J., He S., Shoemaker B.A. (2016). PubChem Substance and Compound Databases. Nucleic Acids Res..

[25] Oduro-Kwateng E., Soliman M.E. (2024). DON/DRP-104 as Potent Serine Protease Inhibitors Implicated in SARS-CoV-2 Infection: Comparative Binding Modes with Human TMPRSS2 and Novel Therapeutic Approach. J. Cell. Biochem..

[26] Daina A., Michielin O., Zoete V. (2017). SwissADME: A Free Web Tool to Evaluate Pharmacokinetics, Drug-Likeness and Medicinal Chemistry Friendliness of Small Molecules. Sci. Rep..

[27] Yao Z.J., Dong J., Che Y.J., Zhu M.F., Wen M., Wang N.N., Wang S., Lu A.P., Cao D.S. (2016). TargetNet: A Web Service for Predicting Potential Drug–Target Interaction Profiling via Multi-Target SAR Models. J. Comput. Aided Mol. Des..

[28] Gallo K., Goede A., Preissner R., Gohlke B.O. (2022). SuperPred 3.0: Drug Classification and Target Prediction—A Machine Learning Approach. Nucleic Acids Res..

[29] Wang X., Shen Y., Wang S., Li S., Zhang W., Liu X., Lai L., Pei J., Li H. (2017). *PharmMapper* 2017 Update: A Web Server for Potential Drug Target Identification with a Comprehensive Target Pharmacophore Database. Nucleic Acids Res..

[30] Breuza L., Poux S., Estreicher A., Famiglietti M.L., Magrane M., Tognolli M., Bridge A., Baratin D., Redaschi N., Xenarios I. (2016). The UniProtKB Guide to the Human Proteome. Database.

[31] Tang Y., Li M., Wang J., Pan Y., Wu F.X. (2015). CytoNCA: A Cytoscape Plugin for Centrality Analysis and Evaluation of Protein Interaction Networks. BioSystems.

[32] Szklarczyk D., Kirsch R., Koutrouli M., Nastou K., Mehryary F., Hachilif R., Gable A.L., Fang T., Doncheva N.T., Pyysalo S. (2023). The STRING Database in 2023: Protein-Protein Association Networks and Functional Enrichment Analyses for Any Sequenced Genome of Interest. Nucleic Acids Res.

[33] Chin C.H., Chen S.H., Wu H.H., Ho C.W., Ko M.T., Lin C.Y. (2014). CytoHubba: Identifying Hub Objects and Sub-Networks from Complex Interactome. BMC Syst. Biol..

[34] Ge S.X., Jung D., Jung D., Yao R. (2020). ShinyGO: A Graphical Gene-Set Enrichment Tool for Animals and Plants. Bioinformatics.

[35] Johnston R.C., Yao K., Kaplan Z., Chelliah M., Leswing K., Seekins S., Watts S., Calkins D., Chief Elk J., Jerome S.V. (2023). Epik: PKa and Protonation State Prediction through Machine Learning. J. Chem. Theory Comput..

[36] Oduro-Kwateng E., Ali M., Kehinde I.O., Zhang Z., Soliman M.E.S. (2024). De Novo Rational Design of Peptide-Based Protein–Protein Inhibitors (Pep-PPIs) Approach by Mapping the Interaction Motifs of the PP Interface and Physicochemical Filtration: A Case on P25-Cdk5-Mediated Neurodegenerative Diseases. J. Cell. Biochem..

[37] Oduro-Kwateng E., Kehinde I.O., Ali M., Kasumbwe K., Mzozoyana V., Parinandi N.L., Soliman M.E.S. (2025). Computational Analysis of Plasmodium Falciparum DNA Damage Inducible Protein 1 (PfDdi1): Insights into Binding of Artemisinin and Its Derivatives and Implications for Antimalarial Drug Design. Cell Biochem. Biophys..

[38] Salmon-Ferrer R., Goetz A.W., Poole D., Le Grand S., Walker R.C. (2013). Routine Microsecond Molecular Dynamics Simulations with AMBER—Part II: Particle Mesh Ewald. J. Chem. Theory Comput..

[39] Mark P., Nilsson L. (2001). Structure and Dynamics of the TIP3P, SPC, and SPC/E Water Models at 298 K. J. Phys. Chem. A.

[40] Roe D.R., Cheatham T.E. (2013). PTRAJ and CPPTRAJ: Software for Processing and Analysis of Molecular Dynamics Trajectory Data. J. Chem. Theory Comput..

[41] Wang E., Sun H., Wang J., Wang Z., Liu H., Zhang J.Z.H., Hou T. (2019). End-Point Binding Free Energy Calculation with MM/PBSA and MM/GBSA: Strategies and Applications in Drug Design. Chem. Rev..

[42] Miller B.R., McGee T.D., Swails J.M., Homeyer N., Gohlke H., Roitberg A.E. (2012). MMPBSA.Py: An Efficient Program for End-State Free Energy Calculations. J. Chem. Theory Comput..

[43] Safran M., Rosen N., Twik M., BarShir R., Stein T.I., Dahary D., Fishilevich S., Lancet D. (2022). The GeneCards Suite. Practical Guide to Life Science Databases.

[44] Mu W., Patankar V., Kitchen S., Zhen A. (2024). Examining Chronic Inflammation, Immune Metabolism, and T Cell Dysfunction in HIV Infection. Viruses.

[45] Moezpoor M.R., Stevenson M. (2024). Help or Hinder: Protein Host Factors That Impact HIV-1 Replication. Viruses.

[46] Hunt P.W. (2010). Th17, Gut, and HIV: Therapeutic Implications. Curr. Opin. HIV AIDS.

[47] Khader S.A., Gaffen S.L., Kolls J.K. (2009). Th17 Cells at the Crossroads of Innate and Adaptive Immunity against Infectious Diseases at the Mucosa. Mucosal Immunol..

[48] Renault C., Veyrenche N., Mennechet F., Bedin A.S., Routy J.P., Van de Perre P., Reynes J., Tuaillon E. (2022). Th17 CD4+ T-Cell as a Preferential Target for HIV Reservoirs. Front. Immunol..

[49] Elhed A., Unutmaz D. (2010). Th17 Cells and HIV Infection. Curr. Opin. HIV AIDS.

[50] O’Shea J.J., Steward-Tharp S.M., Laurence A., Watford W.T., Wei L., Adamson A.S., Fan S. (2009). Signal Transduction and Th17 Cell Differentiation. Microbes Infect..

[51] Waickman A.T., Powell J.D. (2012). MTOR, Metabolism, and the Regulation of T-Cell Differentiation and Function. Immunol. Rev..

[52] Dang E.V., Barbi J., Yang H.Y., Jinasena D., Yu H., Zheng Y., Bordman Z., Fu J., Kim Y., Yen H.R. (2011). Control of TH17/Treg Balance by Hypoxia-Inducible Factor 1. Cell.

[53] Daniels M.A., Teixeiro E. (2025). The NF-ΚB Signaling Network in the Life of T Cells. Front. Immunol..

[54] Haase M., Fitze G. (2016). HSP90AB1: Helping the Good and the Bad. Gene.

[55] Tukaj S., Zillikens D., Kasperkiewicz M. (2014). Inhibitory Effects of Heat Shock Protein 90 Blockade on Proinflammatory Human Th1 and Th17 Cell Subpopulations. J. Inflamm..

[56] Alanazi H.H., Elfaki E. (2023). The Immunomodulatory Role of *Withania somnifera* (L.) Dunal in Inflammatory Diseases. Front. Pharmacol..

[57] Moudgil K.D., Venkatesha S.H. (2023). The Anti-Inflammatory and Immunomodulatory Activities of Natural Products to Control Autoimmune Inflammation. Int. J. Mol. Sci..

[58] Butterfield T.R., Landay A.L., Anzinger J.J. (2020). Dysfunctional Immunometabolism in HIV Infection: Contributing Factors and Implications for Age-Related Comorbid Diseases. Curr. HIV/AIDS Rep..

[59] Lisco A., Vanpouille C., Margolis L. (2009). Coinfecting Viruses as Determinants of HIV Disease. Curr. HIV/AIDS Rep..

[60] Anampa J., Barta S.K., Haigentz M., Sparano J.A. (2019). Human Immunodeficiency Virus (HIV) Infection and Cancer. Abeloff’s Clinical Oncology.

[61] Lin X., Song B., Cao L., Zhang L., Liu S., Wang X., Chen X., Li S. (2025). PD-1 Suppression Enhances HIV Reactivation and T-Cell Immunity via MAPK/NF-ΚB Signaling. Eur. J. Med. Res..

[62] Wang F., Cui Y., Shen X., Wang S., Yang G.B. (2019). IL-17A and IL-17F Repair HIV-1 Gp140 Damaged Caco-2 Cell Barriers by Upregulating Tight Junction Genes. Microbes Infect..

[63] Noorsaeed S., AlBurtamani N., Rokan A., Fassati A. (2025). Heat Shock Protein 90 Is a Chaperone Regulator of HIV-1 Latency. PLoS Pathog..

[64] Mbonye U., Karn J. (2024). The Cell Biology of HIV-1 Latency and Rebound. Retrovirology.

[65] Wang P., Zhang Q., Tan L., Xu Y., Xie X., Zhao Y. (2020). The Regulatory Effects of MTOR Complexes in the Differentiation and Function of CD4+ T Cell Subsets. J. Immunol. Res..

[66] Aiken C., Chen C.H. (2005). Betulinic Acid Derivatives as HIV-1 Antivirals. Trends Mol. Med..

[67] Waki K., Durell S.R., Soheilian F., Nagashima K., Butler S.L., Freed E.O. (2012). Structural and Functional Insights into the HIV-1 Maturation Inhibitor Binding Pocket. PLoS Pathog..

[68] Saisin S., Panthong K., Hongthong S., Kuhakarn C., Thanasansurapong S., Chairoungdua A., Suksen K., Akkarawongsapat R., Napaswad C., Prabpai S. (2023). Pyranonaphthoquinones and Naphthoquinones from the Stem Bark of Ventilago Harmandiana and Their Anti-HIV-1 Activity. J. Nat. Prod..

[69] Tu N.Q., Richetta C., Putzu F., Delelis O., Ahmed K., Masand V.H., Schobert R., Tramontano E., Corona A., Biersack B. (2025). Identification of HIV-1 Reverse Transcriptase-Associated Ribonuclease H Inhibitors Based on 2-Hydroxy-1,4-Naphthoquinone Mannich Bases. Molecules.

[70] Crosby I.T., Bourke D.G., Jones E.D., De Bruyn P.J., Rhodes D., Vandegraaff N., Cox S., Coates J.A.V., Robertson A.D. (2010). Antiviral Agents 2. Synthesis of Trimeric Naphthoquinone Analogues of Conocurvone and Their Antiviral Evaluation against HIV. Bioorg. Med. Chem..

[71] Sandur S.K., Ichikawa H., Sethi G., Kwang S.A., Aggarwal B.B. (2006). Plumbagin (5-Hydroxy-2-Methyl-1,4-Naphthoquinone) Suppresses NF-ΚB Activation and NF-ΚB-Regulated Gene Products through Modulation of P65 and IκBα Kinase Activation, Leading to Potentiation of Apoptosis Induced by Cytokine and Chemotherapeutic Agents. J. Biol. Chem..

[72] Andújar I., Recio M.C., Bacelli T., Giner R.M., Ríos J.L. (2010). Shikonin Reduces Oedema Induced by Phorbol Ester by Interfering with IκBα Degradation Thus Inhibiting Translocation of NF-ΚB to the Nucleus. Br. J. Pharmacol..

[73] Wu H., Dai X., Wang E. (2016). Plumbagin Inhibits Cell Proliferation and Promotes Apoptosis in Multiple Myeloma Cells through Inhibition of the PI3K/Akt-MTOR Pathway. Oncol. Lett..

[74] Hafeez B.B., Jamal M.S., Fischer J.W., Mustafa A., Verma A.K. (2012). Plumbagin, a Plant Derived Natural Agent Inhibits the Growth of Pancreatic Cancer Cells in in Vitro and in Vivo via Targeting EGFR, Stat3 and NF-ΚB Signaling Pathways. Int. J. Cancer.

[75] Kitson R.R.A., Kitsonová D., Siegel D., Ross D., Moody C.J. (2024). Geldanamycin, a Naturally Occurring Inhibitor of Hsp90 and a Lead Compound for Medicinal Chemistry. J. Med. Chem..

