# Supplementary material for: Phytochemicals from Euclea natalensis Modulate Th17 Differentiation, HIV Latency, and Comorbid Pathways: A Systems Pharmacology and Thermodynamic Profiling Approach
Source: Microorganisms. 2025 Sep 15;13(9):2150. doi: 10.3390/microorganisms13092150 (PMC12472539; doi:10.3390/microorganisms13092150)
Supplement: Supplementary file 1 [file microorganisms-13-02150-s001.zip › Supplementary Material 1_Figures.pdf]

Phytochemicals from *Euclea natalensis* Modulate Th17 Differentiation, HIV Latency, and Comorbid Pathways: A Systems Pharmacology and Thermodynamic Profiling Approach

Ernest Oduro-Kwateng [1], Nader E. Abo-Dya [2], Mahmoud E. Soliman [3*], and Nompumelelo P. Mkhwanazi [1*]

[1] HIV Pathogenesis Programme, School of Laboratory Medicine and Medical Sciences, College of Health Science, University of KwaZulu-Natal, South Africa

[2] Department of Pharmaceutical Chemistry, Faculty of Pharmacy, University of Tabuk, Tabuk, 71491, Saudi Arabia

[3] Molecular Bio-computation and Drug Design Research Group, School of Health Sciences, College of Health Science, University of KwaZulu-Natal, South Africa

[*]Corresponding Author: Prof Mahmoud E. Soliman

Email: soliman@ukzn.ac.za

Department of Pharmaceutical Sciences

School of Health Sciences

University of KwaZulu-Natal

Westville Campus

Private Bag X54001

Durban 4000

*Corresponding Author: Dr Nompumelelo P. Mkhwanazi

Email: mkhwanazi@ukzn.ac.za

University of KwaZulu-Natal

College of Health Science

School of Laboratory Medicine and Medical Science

HIV Pathogenesis Programme

DDMRI Building 2nd Floor Room 212

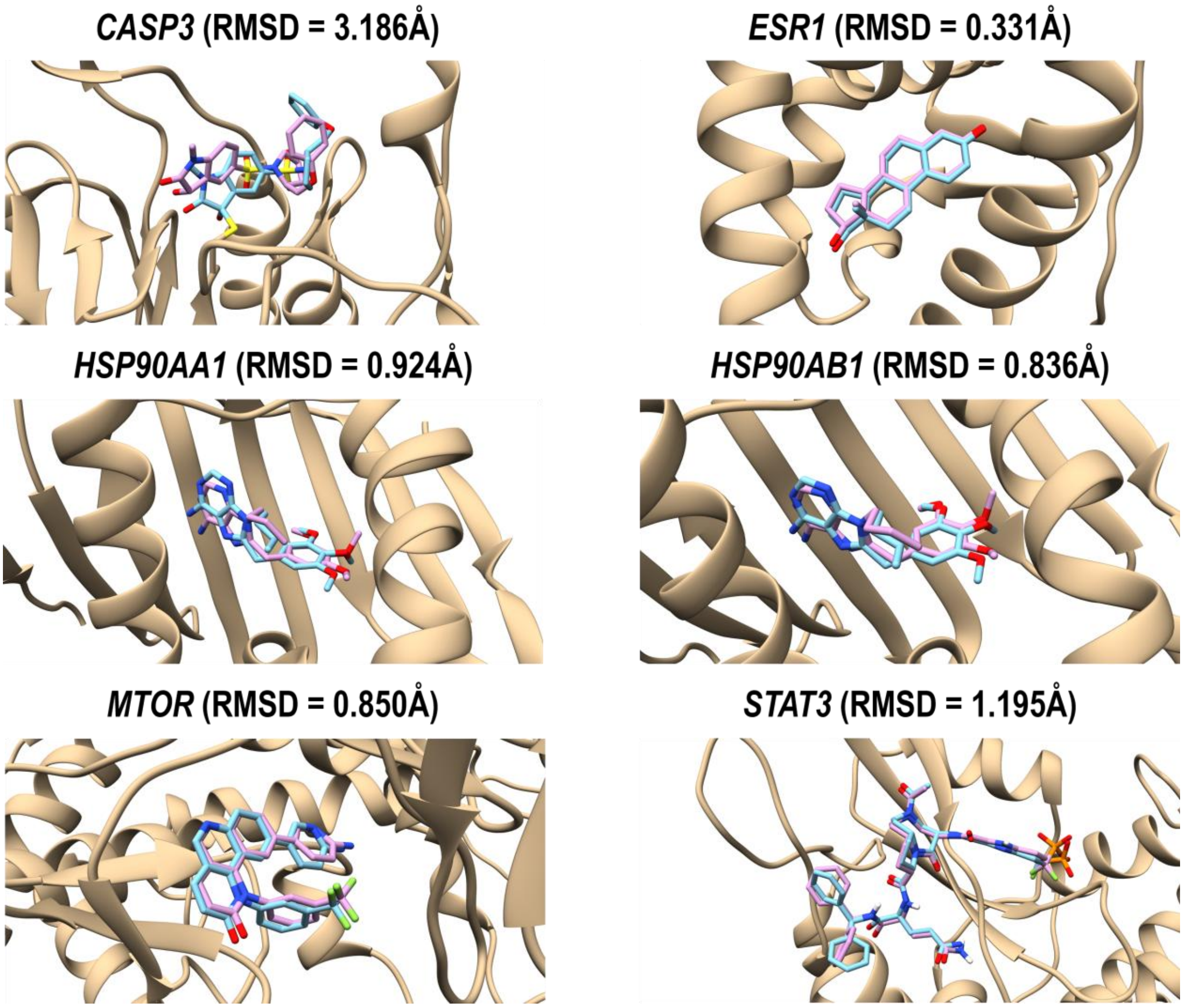

Figure S1. Validation of molecular docking protocol via redocking of co-crystallized ligands using AutoDock Vina. Structural overlays show native (co-crystallized) ligands (sky blue) and their redocked poses (plum) within the binding pockets of HIV-associated protein targets, rendered as tan-colored cartoons. Redocking was performed using AutoDock Vina, and RMSD values were calculated to assess pose reproduction accuracy.

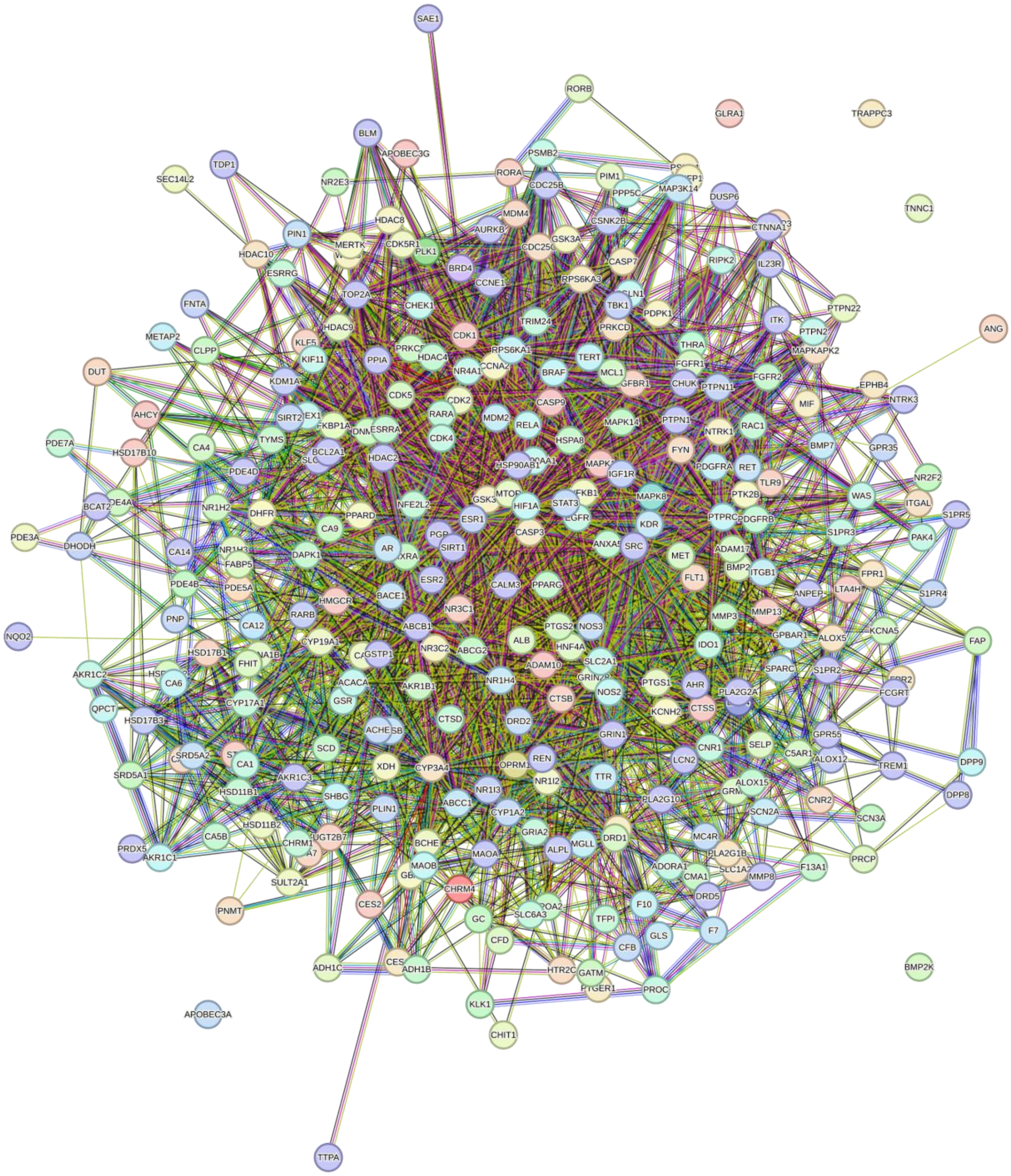

Figure S2. PPI network of overlapping targets between *Euclea Natalensis* phytochemicals and HIV/AIDS-associated genes. The network was generated using the STRING database (confidence score ≥ 0.4), visualizing the interaction map of 313 intersecting targets. Nodes represent individual proteins, while edges denote predicted functional associations. Edge colors indicate the type of interaction evidence: green (gene neighborhood), red (gene fusion), blue (co-occurrence), purple (experimental), yellow (text mining), light blue (database), and black (co-expression). The dense interconnectivity of the network suggests a high degree of

functional cooperation among targets, supporting the potential multi-target therapeutic activity of *E. natalensis* constituents in modulating HIV/AIDS-related host processes.

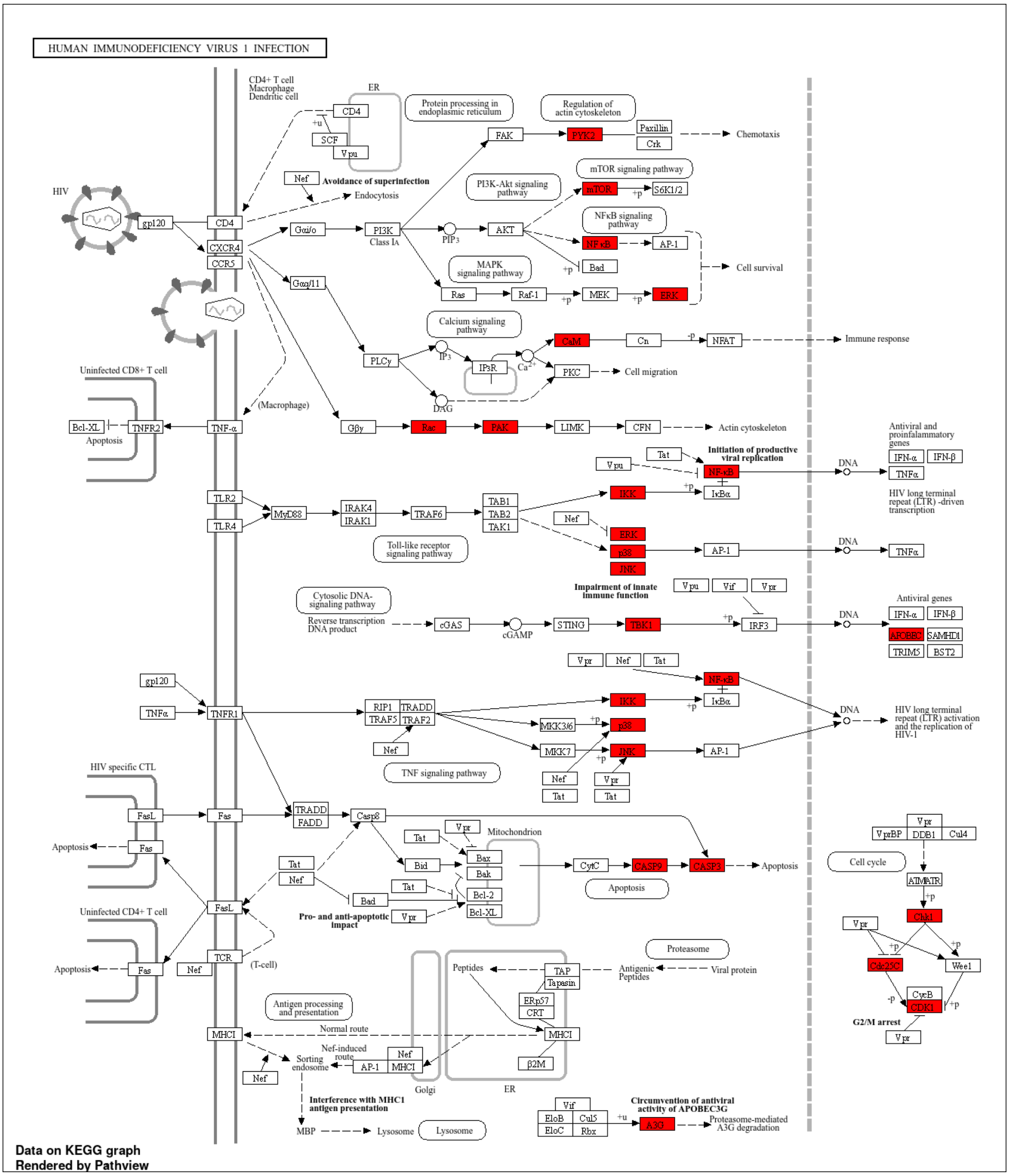

Figure S3. Integrated KEGG map of Human immunodeficiency virus type 1 (HIV-1) infection (hsa05170) highlighting overlapping genes targeted by *Euclea natalensis* phytochemicals. The pathway, rendered via Pathview, illustrates multiple stages of the HIV life cycle, including viral entry, replication, integration, immune evasion, and apoptosis of host cells. Overlapping genes modulated by *E. natalensis* (*PAK4*, *CHEK1*, *CHUK*, *MAPK14*, *PTK2B*, *MTOR*, *TBK1*, *NFκβ1*, *MAPK1*, *MAPK8*, *MAPK10*, *RAC1*, *RELA*, *APOBEC3G*, *CALM1*, *CALM3*, *CASP3*,

CASP9, *CDK1*, and *CDC25C*) are highlighted in red. These genes map to key modules, such as PI3K–Akt–mTOR signaling, NF-κB activation, MAPK cascades, calcium signaling, Toll-like receptor signaling, TNF signaling, and apoptotic pathways, which collectively influence HIV replication dynamics, immune escape, and latency maintenance. Solid arrows indicate direct signaling events, and dashed arrows indicate indirect or inferred interactions. This integrated pathway underscores the potential of the phytochemicals to modulate multiple HIV-1–relevant signaling axes by targeting host immunoregulatory and pro-viral factors.
