# Supplementary material for: Phytochemicals from Euclea natalensis Modulate Th17 Differentiation, HIV Latency, and Comorbid Pathways: A Systems Pharmacology and Thermodynamic Profiling Approach
Source: Microorganisms. 2025 Sep 15;13(9):2150. doi: 10.3390/microorganisms13092150 (PMC12472539; doi:10.3390/microorganisms13092150)
Supplement: Supplementary file 1 [file microorganisms-13-02150-s001.zip › Supplementary Material 2_Tables.pdf]

Phytochemicals from *Euclea natalensis* Modulate Th17 Differentiation, HIV Latency, and Comorbid Pathways: A Systems Pharmacology and Thermodynamic Profiling Approach

Ernest Oduro-Kwateng [1], Nader E. Abo-Dya [2], Mahmoud E. Soliman [3*], and Nompumelelo P. Mkhwanazi [1*]

[1] HIV Pathogenesis Programme, School of Laboratory Medicine and Medical Sciences, College of Health Science, University of KwaZulu-Natal, South Africa

[2] Department of Pharmaceutical Chemistry, Faculty of Pharmacy, University of Tabuk, Tabuk, 71491, Saudi Arabia

[3] Molecular Bio-computation and Drug Design Research Group, School of Health Sciences, College of Health Science, University of KwaZulu-Natal, South Africa

*Corresponding Author: Prof Mahmoud E. Soliman

Email: soliman@ukzn.ac.za

Department of Pharmaceutical Sciences

School of Health Sciences

University of KwaZulu-Natal

Westville Campus

Private Bag X54001

Durban 4000

*Corresponding Author: Dr Nompumelelo P. Mkhwanazi

Email: mkhwanazi@ukzn.ac.za

University of KwaZulu-Natal

College of Health Science

School of Laboratory Medicine and Medical Science

HIV Pathogenesis Programme

DDMRI Building 2nd Floor Room 212

Table S1. Predicted ADME and drug-likeness properties of phytochemicals from *Euclea natalensis* root bark based on SwissADME analysis.

Phytochemical	MW	HBA	HBD	TPSA	iLOGP	ESOL Log S	ESOL Class	GI Abs.	BBB	Pgp	CYP3A4	RO5	B.S	PAINS alerts	SA
20(29)-Lupene -3β-isoferulate	602.89	4	1	55.76	6.23	-10.94	Insoluble	Low	No	No	No	2	0.17	0	6.75
5-hydroxy-4-methoxy -2-nathaldehyde	204.22	3	2	49.69	2.31	-2.66	Soluble	High	Yes	No	No	0	0.55	0	1.48
7-Methyljuglone	188.18	3	1	54.37	1.76	-2.76	Soluble	High	Yes	No	No	0	0.55	1	2.41
8'-hydroxydiospyrin	390.34	7	3	128.97	2.62	-4.99	Moderately soluble	High	No	No	Yes	0	0.55	2	3.39
Betulin	442.72	2	2	40.46	4.47	-7.67	Poorly soluble	Low	No	No	No	1	0.55	0	5.68
Diospyrin	374.34	6	2	108.74	2.53	-4.76	Moderately soluble	High	No	No	Yes	0	0.55	2	3.57
Euclanone	390.34	7	3	128.97	1.12	-4.65	Moderately soluble	High	No	No	Yes	0	0.55	0	3.25
Galpinone	560.51	9	3	163.11	3.21	-6.94	Poorly soluble	Low	No	No	Yes	1	0.55	1	4.24
Isodiospyrin	374.34	6	2	108.74	2.32	-4.76	Moderately soluble	High	No	No	Yes	0	0.55	1	3.36
Lupeol	426.72	1	1	20.23	4.72	-8.64	Poorly soluble	Low	No	No	No	1	0.55	0	5.49
Mamegakinone	374.34	6	2	108.74	2.54	-4.77	Moderately soluble	High	No	No	Yes	0	0.55	1	3.34
Methylnaphthazarin	204.18	4	2	74.6	1.82	-2.8	Soluble	High	No	No	Yes	0	0.55	2	2.47
Natalenone	376.36	6	3	111.9	2.24	-4.28	Moderately soluble	High	No	No	Yes	0	0.55	1	4.42
Neodiospyrin	374.34	6	2	108.74	2.54	-4.76	Moderately soluble	High	No	No	Yes	0	0.55	1	3.57
Octahydroeuclein	382.41	6	4	115.06	2.33	-3.63	Soluble	High	No	Yes	No	0	0.55	0	4.13
Shinanolone	192.21	3	2	57.53	1.81	-2.27	Soluble	High	Yes	No	No	0	0.55	0	2.44
β-sitosterol	414.71	1	1	20.23	5.05	-7.9	Poorly soluble	Low	No	No	No	1	0.55	0	6.3

MW: Molecular weight, HBA: Hydrogen Bond Acceptor, HBD: Hydrogen Bond Donor, TPSA: Topological Polar Surface Area, iLOGP: Lipophilicity, ESOL Log S: Water solubility (log scale), ESOL Class: Water solubility classification, GI Abs.: Gastrointestinal absorption rate, BBB: Blood-Brain Barrier permeability, Pgp: P-glycoprotein substrate status, CYP3A4: Interaction with cytochrome P450 3A4, RO5: Lipinski's Rule of 5 violations, B.S: Bioavailability score, PAINS alerts: Pan Assay Interference Compounds, SA: Synthetic accessibility.

Table S2. UniProt identifiers and gene/protein annotations for the 313 overlapping targets between *Euclea natalensis* phytochemicals and HIV/AIDS-associated genes.

UniProt ID	Protein Name	Gene Name	UniProt ID	Protein Name	Gene Name	UniProt ID	Protein Name	Gene Name	UniProt ID	Protein Name	Gene Name
O43570	CAH12	*CA12*	P30305	MPIP2	*CDC25B*	P03950	ANGI	*ANG*	P00746	CFAD	*CFD*
Q9ULX7	CAH14	*CA14*	P26358	DNMT1	*DNMT1*	P80365	DHI2	*HSD11B2*	P08069	IGF1R	*IGF1R*
P21728	DRD1	*DRD1*	P06401	PRGR	*PGR*	P17706	PTN2	*PTPN2*	P49354	FNTA	*FNTA*
P14174	MIF	*MIF*	P23141	EST1	*CES1*	P05093	CP17A	*CYP17A1*	P11362	FGFR1	*FGFR1*
P16050	LOX15	*ALOX15*	P41235	HNF4A	*HNF4A*	Q96RI1	NR1H4	*NR1H4*	P00742	FA10	*F10*
Q9UNQ0	ABCG2	*ABCG2*	Q9HC16	ABC3G	*APOBEC3G*	P08173	ACM4	*CHRM4*	P06241	FYN	*FYN*
P43166	CAH7	*CA7*	P37058	DHB3	*HSD17B3*	P54132	BLM	*BLM*	P16234	PGFRA	*PDGFRA*
P14061	DHB1	*HSD17B1*	Q99685	MGLL	*MGLL*	P21554	CNR1	*CNR1*	P28335	5HT2C	*HTR2C*
P22748	CAH4	*CA4*	P31941	ABC3A	*APOBEC3A*	P35372	OPRM	*OPRM1*	Q16769	QPCT	*QPCT*
P23280	CAH6	*CA6*	Q9HC97	GPR35	*GPR35*	P34972	CNR2	*CNR2*	Q12884	SEPR	*FAP*
P23219	PGH1	*PTGS1*	P08575	PTPRC	*PTPRC*	Q05655	KPCD	*PRKCD*	P09486	SPRC	*SPARC*
P35869	AHR	*AHR*	P08235	MCR	*NR3C2*	O96013	PAK4	*PAK4*	P11142	HSP7C	*HSPA8*
Q16548	B2LA1	*BCL2A1*	P35398	RORA	*RORA*	O60341	KDM1A	*KDM1A*	P01709	LV208	*IGLV2-8*
P37059	DHB2	*HSD17B2*	Q00987	MDM2	*MDM2*	P40763	STAT3	*STAT3*	P25774	CATS	*CTSS*
P51452	DUS3	*DUSP3*	P04150	GCR	*NR3C1*	P14902	I23O1	*IDO1*	P00390	GSHR	*GSR*
Q92731	ESR2	*ESR2*	P08842	STS	*STS*	Q99250	SCN2A	*SCN2A*	P16109	LYAM3	*SELP*
P55211	CASP9	*CASP9*	P49841	GSK3B	*GSK3B*	P28482	MK01	*MAPK1*	P16083	NQO2	*NQO2*
Q16790	CAH9	*CA9*	P35354	PGH2	*PTGS2*	Q9Y2T6	GPR55	*GPR55*	P04035	HMDH	*HMGCR*
P05177	CP1A2	*CYP1A2*	P15121	ALDR	*AKR1B1*	P56524	HDAC4	*HDAC4*	Q13231	CHIT1	*CHIT1*

P03372	ESR1	*ESR1*	P11388	TOP2A	*TOP2A*	P08709	FA7	*F7*	P07858	CATB	*CTSB*
P21397	AOFA	*MAOA*	P30307	MPIP3	*CDC25C*	Q9H228	S1PR5	*S1PR5*	P15056	BRAF	*BRAF*
Q04206	TF65	*RELA*	P67870	CSK2B	*CSNK2B*	P32245	MC4R	*MC4R*	P18075	BMP7	*BMP7*
P24468	COT2	*NR2F2*	Q00535	CDK5	*CDK5*	Q02127	PYRD	*DHODH*	O15496	PA2GX	*PLA2G10*
Q9NR96	TLR9	*TLR9*	P07900	HS90A	*HSP90AA1*	P41145	OPRK	*OPRK1*	P27487	DPP4	*DPP4*
P05186	PPBT	*ALPL*	Q9GZT9	EGLN1	*EGLN1*	Q9UKV0	HDAC9	*HDAC9*	P49789	FHIT	*FHIT*
Q9Y2D0	CAH5B	*CA5B*	P23415	GLRA1	*GLRA1*	Q05586	NMDZ1	*GRIN1*	P53350	PLK1	*PLK1*
P09917	LOX5	*ALOX5*	P22736	NR4A1	*NR4A1*	O00767	SCD	*SCD*	P00797	RENI	*REN*
P35218	CAH5A	*CA5A*	Q8TDU6	GPBAR	*GPBAR1*	Q96GD4	AURKB	*AURKB*	Q04828	AK1C1	*AKR1C1*
P21918	DRD5	*DRD5*	Q16665	HIF1A	*HIF1A*	Q99558	M3K14	*MAP3K14*	P62508	ERR3	*ESRRG*
P11511	CP19A	*CYP19A1*	Q6V1X1	DPP8	*DPP8*	P25090	FPR2	*FPR2*	P31213	S5A2	*SRD5A2*
P08183	MDR1	*ABCB1*	P55899	FCGRN	*FCGRT*	P11473	VDR	*VDR*	P11229	ACM1	*CHRM1*
O43353	RIPK2	*RIPK2*	P35228	NOS2	*NOS2*	Q969S8	HDA10	*HDAC10*	P33316	DUT	*DUT*
Q02156	KPCE	*PRKCE*	Q06124	PTN11	*PTPN11*	P61964	WDR5	*WDR5*	O60885	BRD4	*BRD4*
Q14432	PDE3A	*PDE3A*	P51812	KS6A3	*RPS6KA3*	Q14289	FAK2	*PTK2B*	P24864	CCNE1	*CCNE1*
P27815	PDE4A	*PDE4A*	P53779	MK10	*MAPK10*	Q12866	MERTK	*MERTK*	P0DP23	CALM1	*CALM1*
O95977	S1PR4	*S1PR4*	P06276	CHLE	*BCHE*	Q99500	S1PR3	*S1PR3*	P08238	HS90B	*HSP90AB1*
P04054	PA21B	*PLA2G1B*	P02768	ALBU	*ALB*	O15111	IKKA	*CHUK*	P10276	RARA	*RARA*
Q01959	SC6A3	*SLC6A3*	P00734	THRB	*F2*	O14672	ADA10	*ADAM10*	O14746	TERT	*TERT*
O95136	S1PR2	*S1PR2*	P02652	APOA2	*APOA2*	Q9HBX9	RXFP1	*RXFP1*	P34995	PE2R1	*PTGER1*
Q9NUW8	TYDP1	*TDP1*	P45983	MK08	*MAPK8*	P23526	SAHH	*AHCY*	P17707	DCAM	*AMD1*
P19838	NFKB1	*NFKB1*	P00918	CAH2	*CA2*	Q13085	ACACA	*ACACA*	O43617	TPPC3	*TRAPPC3*
Q9BY41	HDAC8	*HDAC8*	P09211	GSTP1	*GSTP1*	P04629	NTRK1	*NTRK1*	P49840	GSK3A	*GSK3A*
P07339	CATD	*CTSD*	P00751	CFAB	*CFB*	Q9NY46	SCN3A	*SCN3A*	Q13133	NR1H3	*NR1H3*
P27695	APEX1	*APEX1*	P08254	MMP3	*MMP3*	P10646	TFPI1	*TFPI*	Q14833	GRM4	*GRM4*
O15151	MDM4	*MDM4*	P00915	CAH1	*CA1*	P00374	DYR	*DHFR*	Q9Y2R2	PTN22	*PTPN22*
Q13887	KLF5	*KLF5*	Q16740	CLPP	*CLPP*	P16662	UD2B7	*UGT2B7*	P10826	RARB	*RARB*
O15164	TIF1A	*TRIM24*	P24941	CDK2	*CDK2*	Q9UHD2	TBK1	*TBK1*	P49721	PSB2	*PSMB2*
P11166	GTR1	*SLC2A1*	P78536	ADA17	*ADAM17*	P08684	CP3A4	*CYP3A4*	P07949	RET	*RET*
P21730	C5AR1	*C5AR1*	Q08499	PDE4D	*PDE4D*	P11802	CDK4	*CDK4*	P14416	DRD2	*DRD2*

P10827	THA	*THRA*	O15382	BCAT2	*BCAT2*	P12643	BMP2	*BMP2*	P04070	PROC	*PROC*
P08236	BGLR	*GUSB*	Q16828	DUS6	*DUSP6*	P45452	MMP13	*MMP13*	P42785	PCP	*PRCP*
Q92769	HDAC2	*HDAC2*	P20248	CCNA2	*CCNA2*	P52895	AK1C2	*AKR1C2*	P05556	ITB1	*ITGB1*
Q92753	RORB	*RORB*	P11309	PIM1	*PIM1*	P49137	MAPK2	*MAPKAPK2*	Q13946	PDE7A	*PDE7A*
P22460	KCNA5	*KCNA5*	P62937	PPIA	*PPIA*	O14757	CHK1	*CHEK1*	Q12809	KCNH2	*KCNH2*
Q99714	HCD2	*HSD17B10*	Q00975	CAC1B	*CACNA1B*	P20701	ITAL	*ITGAL*	P00326	ADH1G	*ADH1C*
P42262	GRIA2	*GRIA2*	Q9Y5X4	NR2E3	*NR2E3*	P56817	BACE1	*BACE1*	P08581	MET	*MET*
P15144	AMPN	*ANPEP*	P47989	XDH	*XDH*	P54760	EPHB4	*EPHB4*	P35221	CTNA1	*CTNNA1*
Q16236	NF2L2	*NFE2L2*	P30542	AA1R	*ADORA1*	P55210	CASP7	*CASP7*	P11474	ERR1	*ESRRA*
Q86TI2	DPP9	*DPP9*	P35968	VGFR2	*KDR*	Q06520	ST2A1	*SULT2A1*	P14555	PA2GA	*PLA2G2A*
Q5VWK5	IL23R	*IL23R*	Q9UBE0	SAE1	*SAE1*	P63316	TNNC1	*TNNC1*	Q01469	FABP5	*FABP5*
O75469	NR1I2	*NR1I2*	P33527	MRP1	*ABCC1*	P36897	TGFR1	*TGFBR1*	P22894	MMP8	*MMP8*
P43003	EAA1	*SLC1A3*	P17948	VGFR1	*FLT1*	P29474	NOS3	*NOS3*	P21802	FGFR2	*FGFR2*
P18054	LOX12	*ALOX12*	P09619	PGFRB	*PDGFRB*	Q07343	PDE4B	*PDE4B*	P55055	NR1H2	*NR1H2*
P06870	KLK1	*KLK1*	Q9NP99	TREM1	*TREM1*	P49638	TTPA	*TTPA*	P53355	DAPK1	*DAPK1*
O94925	GLSK	*GLS*	P18031	PTN1	*PTPN1*	P00325	ADH1B	*ADH1B*	Q8IXJ6	SIR2	*SIRT2*
Q16288	NTRK3	*NTRK3*	P80188	NGAL	*LCN2*	P42768	WASP	*WAS*	Q96EB6	SIR1	*SIRT1*
P20618	PSB1	*PSMB1*	P42330	AK1C3	*AKR1C3*	P53041	PPP5	*PPP5C*	P19634	SL9A1	*SLC9A1*
P52732	KIF11	*KIF11*	P42574	CASP3	*CASP3*	Q13526	PIN1	*PIN1*	Q15418	KS6A1	*RPS6KA1*
P30044	PRDX5	*PRDX5*	P23946	CMA1	*CMA1*	Q9NSY1	BMP2K	*BMP2K*	P62942	FKB1A	*FKBP1A*
P02766	TTHY	*TTR*	Q14994	NR1I3	*NR1I3*	P00488	F13A	*F13A1*	Q13224	NMDE2	*GRIN2B*
P50579	MAP2	*METAP2*	P04818	TYSY	*TYMS*	P06493	CDK1	*CDK1*	P18405	S5A1	*SRD5A1*
P50440	GATM	*GATM*	P37231	PPARG	*PPARG*	P04062	GBA1	*GBA1*	P22303	ACES	*ACHE*
P12931	SRC	*SRC*	P04278	SHBG	*SHBG*	Q03181	PPARD	*PPARD*	P21462	FPR1	*FPR1*
P10275	ANDR	*AR*	P19793	RXRA	*RXRA*	P00491	PNPH	*PNP*	Q08881	ITK	*ITK*
Q07820	MCL1	*MCL1*	P00533	EGFR	*EGFR*	P08758	ANXA5	*ANXA5*	P42345	MTOR	*MTOR*
P27338	AOFB	*MAOB*	Q16539	MK14	*MAPK14*	O15530	PDPK1	*PDPK1*	P11086	PNMT	*PNMT*
O00748	EST2	*CES2*	P02774	VTDB	*GC*	Q15078	CD5R1	*CDK5R1*			
O60240	PLIN1	*PLIN1*	O76074	PDE5A	*PDE5A*	P28845	DHI1	*HSD11B1*			
P63000	RAC1	*RAC1*	P09960	LKHA4	*LTA4H*	O76054	S14L2	*SEC14L2*			

Table S3. The top 20 enriched GO biological processes (BP) associated with the hub genes targeted by *Euclea natalensis* phytochemicals.

Rank	Pathway Name	Pathway ID	Enrichment FDR	Fold Enrichment	Pathway Genes	Hub Genes	Hub Gene Enrichment
1	Reg. of glycolytic proc.	GO:0006110	7.94×10^{-05}	143.01	48	3	*HIF1A MTOR STAT3*
2	Response to osmotic stress	GO:0006970	7.12×10^{-06}	99.48	92	4	*MTOR HSP90AA1 HSP90AB1 CASP3*
3	Reg. of carbohydrate metabolic proc.	GO:0006109	7.61×10^{-05}	49.21	186	4	*HIF1A MTOR NFKB1 STAT3*
4	Negative reg. of apoptotic proc.	GO:0043066	5.48×10^{-06}	16.61	964	7	*MTOR HIF1A ALB HSP90AB1 CASP3 NFKB1 ANXA5*
5	Negative reg. of programmed cell death	GO:0043069	5.48×10^{-06}	16.23	987	7	*MTOR HIF1A ALB HSP90AB1 CASP3 NFKB1 ANXA5*
6	Response to hormone	GO:0009725	6.20×10^{-05}	15.29	898	6	*ESR1 STAT3 HSP90AA1 CASP3 MTOR NFKB1*
7	Negative reg. of cell death	GO:0060548	8.63×10^{-06}	14.40	1112	7	*MTOR HIF1A ALB HSP90AB1 CASP3 NFKB1 ANXA5*
8	Reg. of apoptotic proc.	GO:0042981	3.61×10^{-07}	12.92	1594	9	*CASP3 MTOR HSP90AA1 HIF1A ALB ESR1 HSP90AB1 NFKB1 ANXA5*
9	Reg. of programmed cell death	GO:0043067	3.61×10^{-07}	12.66	1627	9	*CASP3 MTOR HSP90AA1 HIF1A ALB ESR1 HSP90AB1 NFKB1 ANXA5*
10	Reg. of cell death	GO:0010941	6.24×10^{-07}	11.38	1810	9	*CASP3 MTOR HSP90AA1 HIF1A ALB ESR1 HSP90AB1 NFKB1 ANXA5*
11	Response to oxygen-containing compound	GO:1901700	7.01×10^{-06}	10.45	1752	8	*STAT3 ESR1 HIF1A MTOR HSP90AA1 HSP90AB1 NFKB1 CASP3*
12	Apoptotic proc.	GO:0006915	1.52×10^{-06}	9.97	2065	9	*CASP3 MTOR HSP90AA1 HIF1A ALB ESR1 HSP90AB1 NFKB1 ANXA5*
13	Programmed cell death	GO:0012501	1.58×10^{-06}	9.68	2127	9	*CASP3 MTOR HSP90AA1 HIF1A ALB ESR1 HSP90AB1 NFKB1 ANXA5*

14	Cell death	GO:0008219	2.87×10^{-06}	8.88	2320	9	*CASP3 MTOR HSP90AA1 HIF1A ALB ESR1 HSP90AB1 NFKB1 ANXA5*
15	Reg. of catalytic activity	GO:0050790	5.48×10^{-06}	7.98	2582	9	*HSP90AB1 HSP90AA1 NFKB1 HIF1A ANXA5 CASP3 MTOR STAT3 ESR1*
16	Response to organic substance	GO:0010033	2.27×10^{-05}	6.30	3269	9	*ESR1 STAT3 HIF1A HSP90AB1 CASP3 MTOR HSP90AA1 NFKB1 ANXA5*
17	Reg. of molecular function	GO:0065009	2.69×10^{-05}	6.14	3356	9	*HSP90AB1 HSP90AA1 ESR1 NFKB1 HIF1A ANXA5 CASP3 MTOR STAT3*
18	Reg. of biological quality	GO:0065008	5.77×10^{-06}	5.58	4103	10	*ALB HSP90AA1 HSP90AB1 CASP3 HIF1A NFKB1 ESR1 ANXA5 STAT3 MTOR*
19	Response to stress	GO:0006950	8.76×10^{-06}	5.17	4424	10	*HSP90AA1 HSP90AB1 HIF1A STAT3 MTOR ALB ESR1 NFKB1 ANXA5 CASP3*
20	Response to chemical	GO:0042221	1.93×10^{-05}	4.75	4821	10	*ESR1 HIF1A STAT3 MTOR HSP90AB1 CASP3 HSP90AA1 NFKB1 ALB ANXA5*

Table S4. The top 20 enriched GO cellular components (CC) associated with the hub genes targeted by *Euclea natalensis* phytochemicals.

Rank	Pathway Name	Pathway ID	Enrichment FDR	Fold Enrichment	Pathway Genes	Hub Genes	Hub Gene Enrichment
1	Ooplasm	GO:1990917	9.10×10^{-03}	762.70	3	1	*HSP90AB1*
2	Dendritic growth cone	GO:0044294	4.10×10^{-04}	508.47	9	2	*HSP90AA1 HSP90AB1*
3	Dendrite terminus	GO:0044292	4.10×10^{-04}	326.87	14	2	*HSP90AA1 HSP90AB1*
4	Sperm plasma membrane	GO:0097524	5.10×10^{-04}	269.19	17	2	*HSP90AA1 HSP90AB1*
5	Axonal growth cone	GO:0044295	9.70×10^{-04}	183.05	25	2	*HSP90AA1 HSP90AB1*
6	Brush border membrane	GO:0031526	3.70×10^{-03}	78.90	58	2	*HSP90AA1 HSP90AB1*
7	Euchromatin	GO:0000791	4.20×10^{-03}	70.40	65	2	*ESR1 HIF1A*
8	Brush border	GO:0005903	9.00×10^{-03}	40.50	113	2	*HSP90AA1 HSP90AB1*
9	Melanosome	GO:0042470	9.00×10^{-03}	37.51	122	2	*HSP90AA1 HSP90AB1*
10	Pigment granule	GO:0048770	9.00×10^{-03}	37.51	122	2	*HSP90AA1 HSP90AB1*
11	Secretory granule lumen	GO:0034774	4.10×10^{-04}	24.87	368	4	*HSP90AA1 HSP90AB1 NFKB1 ALB*
12	Cytoplasmic vesicle lumen	GO:0060205	4.10×10^{-04}	24.67	371	4	*HSP90AA1 HSP90AB1 NFKB1 ALB*
13	Vesicle lumen	GO:0031983	4.10×10^{-04}	24.54	373	4	*HSP90AA1 HSP90AB1 NFKB1 ALB*
14	Neuronal cell body	GO:0043025	9.00×10^{-03}	13.38	513	3	*HSP90AA1 HSP90AB1 CASP3*
15	Transcription regulator complex	GO:0005667	9.70×10^{-03}	12.41	553	3	*STAT3 HIF1A ESR1*
16	Somatodendritic compartment	GO:0036477	4.30×10^{-03}	10.31	888	4	*HSP90AA1 HSP90AB1 CASP3 MTOR*
17	Secretory granule	GO:0030141	6.00×10^{-03}	9.27	987	4	*HSP90AA1 HSP90AB1 NFKB1 ALB*
18	Secretory vesicle	GO:0099503	9.00×10^{-03}	7.86	1165	4	*HSP90AA1 HSP90AB1 NFKB1 ALB*
19	Nucleoplasm	GO:0005654	1.30×10^{-03}	4.00	4581	8	*HIF1A NFKB1 STAT3 ESR1 MTOR HSP90AA1 HSP90AB1 CASP3*

20	Nuclear lumen	GO:0031981	2.10 × 10^{-03}	3.68	4973	8	*HIF1A NFKB1 STAT3 ESR1 MTOR HSP90AA1 HSP90AB1 CASP3*

Table S5. The top 20 enriched GO molecular functions (MF) associated with the hub genes targeted by *Euclea natalensis* phytochemicals.

Rank	Pathway Name	Pathway ID	Enrichment FDR	Fold Enrichment	Pathway Genes	Hub Genes	Hub Gene Enrichment
1	UTP binding	GO:0002134	2.50 × 10^{-05}	1525.40	3	2	*HSP90AA1 HSP90AB1*
2	Sulfonylurea receptor binding	GO:0017098	2.50 × 10^{-05}	1525.40	3	2	*HSP90AA1 HSP90AB1*
3	Adenyl deoxyribonucleotide binding	GO:0032558	2.50 × 10^{-05}	1525.40	3	2	*HSP90AA1 HSP90AB1*
4	Purine deoxyribonucleotide binding	GO:0032554	3.30 × 10^{-05}	1144.05	4	2	*HSP90AA1 HSP90AB1*
5	Pyrimidine ribonucleotide binding	GO:0032557	3.30 × 10^{-05}	1144.05	4	2	*HSP90AA1 HSP90AB1*
6	Nitric-oxide synthase regulator activity	GO:0030235	4.00 × 10^{-07}	980.61	7	3	*HSP90AA1 HSP90AB1 ESR1*
7	Deoxyribonucleotide binding	GO:0032552	4.70 × 10^{-05}	915.24	5	2	*HSP90AA1 HSP90AB1*
8	TPR domain binding	GO:0030911	6.20 × 10^{-05}	762.70	6	2	*HSP90AA1 HSP90AB1*
9	Pyrimidine nucleotide binding	GO:0019103	7.70 × 10^{-05}	653.74	7	2	*HSP90AA1 HSP90AB1*
10	DNA polymerase binding	GO:0070182	4.50 × 10^{-04}	228.81	20	2	*HSP90AA1 HSP90AB1*
11	Disordered domain specific binding	GO:0097718	1.10 × 10^{-03}	123.68	37	2	*HSP90AA1 HSP90AB1*
12	Histone deacetylase binding	GO:0042826	3.50 × 10^{-04}	50.85	135	3	*HIF1A HSP90AA1 HSP90AB1*
13	DNA-binding transcription activator activity RNA polymerase II-specific	GO:0001228	4.70 × 10^{-04}	18.87	485	4	*ESR1 HIF1A NFKB1 STAT3*
14	DNA-binding transcription activator activity	GO:0001216	4.70 × 10^{-04}	18.72	489	4	*ESR1 HIF1A NFKB1 STAT3*
15	Protein kinase binding	GO:0019901	1.50 × 10^{-04}	15.48	739	5	*HSP90AB1 STAT3 HSP90AA1 ESR1 HIF1A*
16	Transcription factor binding	GO:0008134	1.10 × 10^{-03}	14.32	639	4	*MTOR ESR1 HIF1A STAT3*
17	Kinase binding	GO:0019900	2.10 × 10^{-04}	13.88	824	5	*HSP90AB1 STAT3 HSP90AA1 ESR1 HIF1A*

18	Cis-regulatory region sequence-specific DNA binding	GO:0000987	1.10×10^{-03}	8.95	1278	5	*ESR1 NFKB1 STAT3 MTOR HIF1A*
19	Identical protein binding	GO:0042802	1.80×10^{-04}	6.84	2342	7	*HSP90AA1 HSP90AB1 NFKB1 STAT3 ESR1 ALB MTOR*
20	Nucleic acid binding	GO:0003676	6.50×10^{-04}	4.16	4400	8	*HSP90AA1 HSP90AB1 ESR1 HIF1A NFKB1 STAT3 ALB MTOR*

Table S6. The top 20 enriched KEGG pathways associated with hub genes targeted by *Euclea natalensis* phytochemicals.

Rank	Pathway Name	Pathway ID	Enrichment FDR	Fold Enrichment	Pathway Genes	Hub Genes	Hub Gene Enrichment
1	Th17 cell differentiation	hsa04659	2.09×10^{-10}	127.12	108	6	*MTOR HIF1A HSP90AA1 HSP90AB1 NFKB1 STAT3*
2	PD-L1 expression and PD-1 checkpoint pathway in cancer	hsa05235	7.75×10^{-07}	102.84	89	4	*MTOR HIF1A NFKB1 STAT3*
3	Acute myeloid leukemia	hsa05221	2.49×10^{-05}	102.45	67	3	*MTOR NFKB1 STAT3*
4	Adipocytokine signaling pathway	hsa04920	2.49×10^{-05}	99.48	69	3	*MTOR NFKB1 STAT3*
5	IL-17 signaling pathway	hsa04657	8.10×10^{-07}	98.41	93	4	*HSP90AA1 HSP90AB1 NFKB1 CASP3*
6	Prolactin signaling pathway	hsa04917	2.49×10^{-05}	98.06	70	3	*ESR1 NFKB1 STAT3*
7	Prostate cancer	hsa05215	8.54×10^{-07}	94.35	97	4	*MTOR HSP90AA1 HSP90AB1 NFKB1*
8	Pancreatic cancer	hsa05212	3.01×10^{-05}	90.32	76	3	*MTOR NFKB1 STAT3*
9	HIF-1 signaling pathway	hsa04066	1.23×10^{-06}	83.97	109	4	*MTOR HIF1A NFKB1 STAT3*
10	Chemical carcinogenesis-receptor activation	hsa05207	3.16×10^{-09}	69.69	197	6	*ESR1 MTOR HSP90AA1 HSP90AB1 NFKB1 STAT3*
11	AGE-RAGE signaling pathway in diabetic complications	hsa04933	6.50×10^{-05}	68.64	100	3	*NFKB1 STAT3 CASP3*
12	Insulin resistance	hsa04931	7.37×10^{-05}	63.56	108	3	*MTOR NFKB1 STAT3*
13	Kaposi sarcoma-associated herpesvirus infection	hsa05167	3.05×10^{-07}	58.97	194	5	*MTOR HIF1A NFKB1 STAT3 CASP3*

14	MicroRNAs in cancer	hsa05206	5.37×10^{-06}	56.85	161	4	*MTOR NFKB1 STAT3 CASP3*
15	Proteoglycans in cancer	hsa05205	3.05×10^{-07}	56.64	202	5	*ESR1 MTOR HIF1A STAT3 CASP3*
16	Lipid and atherosclerosis	hsa05417	3.40×10^{-07}	53.46	214	5	*HSP90AA1 HSP90AB1 NFKB1 STAT3 CASP3*
17	Human cytomegalovirus infection	hsa05163	1.84×10^{-05}	40.86	224	4	*MTOR NFKB1 STAT3 CASP3*
18	Salmonella infection	hsa05132	2.49×10^{-05}	36.76	249	4	*HSP90AA1 HSP90AB1 NFKB1 CASP3*
19	Pathways in cancer	hsa05200	2.09×10^{-10}	34.54	530	8	*ESR1 MTOR HIF1A HSP90AA1 HSP90AB1 NFKB1 STAT3 CASP3*
20	PI3K-Akt signaling pathway	hsa04151	7.11×10^{-05}	25.85	354	4	*MTOR HSP90AA1 HSP90AB1 NFKB1*

Table S7. Topological parameters of key nodes, including hub genes and *Euclea natalensis* phytochemicals, in the BA-TAR-PATH network construction, ranked by degree centrality.

Rank	Node	Degree Centrality (DC)	Betweenness Centrality (BC)	Closeness Centrality (CC)
1	*NFKB1*	27	492.572	0.625
2	*STAT3*	20	216.910	0.523
3	*ESR1*	20	260.456	0.523
4	*HSP90AA1*	19	208.184	0.511
5	*MTOR*	16	170.569	0.479
6	*CASP3*	16	128.199	0.479
7	*HIF1A*	15	127.832	0.469
8	*HSP90AB1*	12	63.595	0.441
9	*ALB*	11	56.419	0.433
10	*ANXA5*	9	27.264	0.417
11	Natalenone	8	56.222	0.536
12	Mamegakinone	8	47.917	0.536
13	Octahydroeuclein	7	36.369	0.523
14	Neodiospyrin	7	27.985	0.479
15	β-sitosterol	6	41.511	0.511
16	Diospyrin	6	18.477	0.469
17	Isodiospyrin	5	12.487	0.441
18	Galpinone	5	12.408	0.459
19	Shinanolone	4	10.539	0.489
20	Lupeol	4	14.269	0.489
21	Euclanone	4	6.614	0.425
22	Betulin	4	14.269	0.489
23	7-Methyljuglone	4	6.791	0.425
24	Methylnaphthazarin	3	3.338	0.395
25	8'-hydroxydiospyrin	3	3.885	0.402
26	5-hydroxy-4-methoxy-2-nathaldehyde	2	2.810	0.469

Table S8. Protein targets, PDB IDs, resolutions, and docking coordinates of the hub genes in the BA-TAR-PATH network analysis selected for molecular docking.

Protein Target	PDB ID	Resolution (Å)	Docking Coordinates (Center, Size)		
			X	Y	Z
NFKB1	1SVC	2.60	34.5323, 18.3183	12.1981, 17.9413	36.7446, 20.6011
STAT3	6NUQ	3.15	9.43519, 21.5095	55.1667, 34.6193	3.1702, 23.5683
ESR1	1A52	2.80	105.6869, 18.7388	15.8967, 27.9858	96.5363, 24.5868

HSP90AA1	6N8X	1.49	4.3654, 30.0249	14.1707, 20.6646	220.0280, 20.7412
MTOR	4JSX	3.50	51.2863, 24.8771	-0.9786, 22.3873	-45.7329, 23.9441
CASP3	1GFW	2.80	37.7474, 23.9127	34.0327, 18.9778	28.9805, 22.2833
HIF1A	4ZPR	3.90	-108.4180, 54.3949	-44.2623, 44.6041	3.0718, 53.4124
HSP90AB1	6N8Y	1.55	-4.3423, 28.9924	-13.5388, 19.5826	-23.2978, 20.5741

Table S9. Predicted ionization states and pKa values for the 8 key *E. natalensis* phytochemicals at physiological pH (7.4 ± 0.5), calculated using Epik (Schrödinger Suite 2023-2). For each ionizable atom, the predicted acidic or basic pKa, penalty (kcal/mol), and most probable net charge are reported.

Compound	Atom	Acid/Base	Predicted pKa	Penalty (kcal/mol)	Net Charge
Diospyrin	O1H	Conjugate acid	7.76	0.22	0
	O2	Conjugate base	5.85		
	O3H	Conjugate acid	8.79		
	O4	Conjugate base	3.63		
	O5	Conjugate base	4.3		
	O6	Conjugate base	5.37		
	C12	Conjugate base	4.61		
	C14	Conjugate base	4.9		
Galpinone	O1H	Conjugate acid	8.18	0.1	0
	O2	Conjugate base	5.47		
	O3H	Conjugate acid	8.51		
	O4	Conjugate base	4.51		
	O5	Conjugate base	5.41		
	O6	Conjugate base	5.48		
	O7	Conjugate base	4.21		
	O8H	Conjugate acid	8.99		
	O9	Conjugate base	4.4		
Isodiospyrin	O1H	Conjugate acid	8.08	0.12	0
	O2H	Conjugate acid	8.51		
	O3	Conjugate base	5.41		
	O4	Conjugate base	4.21		
	O5	Conjugate base	4.34		
	O6	Conjugate base	5.39		

	C13	Conjugate base	5.05		
	C14	Conjugate base	4.97		
Mamegakinone	O1	Conjugate base	5.48	0.02	0
	O2	Conjugate base	5.48		
	O3H	Conjugate acid	8.99		
	O4H	Conjugate acid	8.99		
	O5	Conjugate base	4.4		
	O6	Conjugate base	4.4		
	C9	Conjugate base	4.85		
	C10	Conjugate base	4.85		
Natalenone	C1	Conjugate base	4.34	0.04	0
	O11	Conjugate base	4.78		
	O12	Conjugate base	5.94		
	O13H	Conjugate acid	8.59		
	O18H	Conjugate acid	10.38		
	C23	Conjugate base	4.31		
	O29H	Conjugate acid	9.03		
	O30	Conjugate base	4.8		
Neodiospyrin	O1H	Conjugate acid	8.38	0.06	0
	O2	Conjugate base	4.97		
	O3H	Conjugate acid	8.96		
	O4	Conjugate base	4.37		
	O5	Conjugate base	5.23		
	O6	Conjugate base	4.16		
	C11	Conjugate base	4.53		
	C12	Conjugate base	5.34		
Octahydroeuclein	O1H	Conjugate acid	12.4	0.01	0
	O2H	Conjugate acid	9.34		
	O4	Conjugate base	4.23		
	O5H	Conjugate acid	9.27		
	O6	Conjugate base	5.31		
	C14	Conjugate base	8.28		
β-sitosterol				0	0

Table S10. Thermodynamic descriptors of protein–ligand complexes based on 200 ns MD simulations.

MD System	RMSD (Å)	RMSF (Å)	RoG (Å)	SASA (Å^2)
HSP90AA1	1.53±0.22	0.89±0.62	17.17±0.08	10775.99±236.70
HSP90AA1–Diospyrin	1.67±0.34	0.92±0.67	16.97±0.08	10467.41±247.75
HSP90AA1– PU-11	1.64±0.38	0.88±0.88	17.01±0.08	10467.00±250.50
HSP90AB1	3.99±0.76	2.42±2.02	19.19±0.37	12410.69±272.49
HSP90AB1–Diospyrin	3.02±0.63	1.69±1.86	18.28±0.35	12021.46±418.44
HSP90AB1– PU-11	6.33±1.25	1.31±1.20	19.00±0.17	12442.97±266.36
NFκβ1	2.90±0.97	2.29±0.97	24.83±0.31	15640.04±363.24
NFκβ1– Galpinone	3.67±1.07	1.72±0.73	24.36±0.44	14776.18±357.74
NFκβ1–IMD 0354	5.25±0.96	1.66±0.65	25.95±0.21	15350.44±321.82
MTOR	3.74±0.53	1.41±0.70	35.88±0.13	59263.54±638.74
MTOR–Galpinone	4.51±1.09	1.84±1.35	35.59±0.20	59492.33±774.85
MTOR–Torin 2	4.16±0.99	1.91±1.27	36.25±0.22	58615.60±798.19
STAT3	2.70±0.42	1.36±0.87	35.58±0.18	27414.40±406.99
STAT3–Galpinone	2.69±0.29	1.41±0.91	35.68±0.21	27681.33±439.07
STAT3–SI-109	3.30±0.69	1.56±1.40	35.49±0.16	27434.97±404.80

Root mean square deviation (RMSD), root mean square fluctuation (RMSF), radius of gyration (RoG), and solvent accessible surface area (SASA) values (mean ± standard deviation) are reported for apo and ligand-bound forms of HIV-associated host proteins.
